# Supplementary material for: New Building Blocks for Self-Healing Polymers
Source: Polymers (Basel). 2022 Dec 9;14(24):5394. doi: 10.3390/polym14245394 (PMC9784872; doi:10.3390/polym14245394)
Supplement: Supplementary file 1 [file polymers-14-05394-s001.zip › polymers-2071942-SI.pdf]

Supporting information

New building-blocks for self-healing polymers

Elena O. Platonova *[a], Polina Ponomareva [a], Zalina Lokiaeva [a], Alexander A. Pavlov [b] and Alexander V. Polezhaev *[a, b]

[a]*N.E. Bauman Moscow State Technical University, 105005, Russia, Moscow, 2nd Baumanskaya str., 5/1*

[b]*A.N. Nesmeyanov Institute of Organoelement Compounds, 119334, Russia, Moscow, Vavilova str., 28*

*Correspondence to: A. Polezhaev (E-mail: aleksandr.polejaev@gmail.com), E. Platonova (E-mail: EPlatonova@emtc.ru).

Table of Contents

Experimental Section
1. Materials
2. Synthesis and sample preparation
2.1 Synthesis of DFA-T
2.2 Synthesis of FA-T
2.3 Synthesis of FOH-T
2.4 Synthesis of DFA-M
2.5 Synthesis of FA-M
2.6 Synthesis of FOH-M
2.7 Synthesis of DFA-H
2.8 Synthesis of FA-H
2.9 Synthesis of FOH-H
2.10 Synthesis of linear polyurethane PU-HEMI
2.11 Synthesis of PU-DA.
2.12 Sample preparation of PU-DA
2.13 Self-healing ability test of PU-DA
3. Characterization
3.1 Nuclear Magnetic Resonance (NMR)
Figure S1 ^{1}H NMR spectrum of DFA-T in DMSO-d^{6}.
Figure S2 ^{13}C NMR spectrum of DFA-T in DMSO-d^{6}
Figure S3 ^{1}H NMR spectrum of FA-T in DMSO-d^{6}.
Figure S4 ^{13}C NMR spectrum of FA-T in DMSO-d^{6}
Figure S5 ^{1}H NMR spectrum of FOH-T in DMSO-d^{6}.
Figure S6 ^{13}C NMR spectrum of FOH-T in DMSO-d^{6}.
Figure S7 ^{1}H NMR spectrum of DFA-M in DMSO-d^{6}.
Figure S8 ^{13}C NMR spectrum of DFA-M in DMSO-d^{6}.
Figure S9 ^{1}H NMR spectrum of FA-M in DMSO-d^{6}.
Figure S10 ^{13}C NMR spectrum of FA-M in DMSO-d^{6}
Figure S11 ^{1}H NMR spectrum of FOH-M in DMSO-d^{6}.
Figure S12 ^{13}C NMR spectrum of FOH-M in DMSO-d^{6}
Figure S13 ^{1}H NMR spectrum of DFA-H in DMSO-d^{6}.
Figure S14 ^{13}C NMR spectrum of DFA-H in DMSO-d^{6}
Figure S15 ^{1}H NMR spectrum of FA-H in DMSO-d^{6}.
Figure S16 ^{13}C NMR spectrum of FA-H in DMSO-d^{6}
Figure S17 ^{1}H NMR spectrum of FOH-H in DMSO-d^{6}.
Figure S18 ^{13}C NMR spectrum of FOH-H in DMSO-d^{6}
Figure S19 ^{1}H NMR spectrum of PU prepolymer in DMSO-d^{6}.
Figure S20 ^{13}C NMR spectrum of PU prepolymer in DMSO-d^{6}.
Figure S21 ^{1}H NMR spectrum of PU-DA in DMSO-d^{6}.
Figure S22 ^{13}C NMR spectrum of PU-DA in DMSO-d^{6}.
3.2 Attenuated Total Reflection-Fourier Transform Infrared Spectroscopy (ATR-FTIR)
3.3 Differential Scanning Calorimetry (DSC)
Table S1 Melting points of furan-containing ureas
Figure S23 DSC curves of the T-series ureas.
Figure S24 DSC curves of the M-series ureas.
Figure S25 DSC curves of the H-series ureas.
Figure S26 DSC curves of the PU prepolymer.
Figure S27 DSC curves of PU-DA.
3.4 Thermogravimetric Analysis (TGA)
Figure S28 TGA and DTG curves of PU-DA.
3.5 Gel permeation chromatography (GPC)
Figure S29 GPC chromatograms

3.6 Quantification of self-healing process efficiency
Figure S30 Shape of tensile strength samples of PU-DA.
Table S2. Tensile test parameters.

Experimental Section

1. Materials

DFA (difurfurylamine) was prepared by modified method [1]. N-(2-Hydroxyethyl)-maleimide (HEMI) was synthesized via literary method [2]. MDI (methylene diphenyl diisocyanate, 98%), HDI (hexamethylene diisocyanate, 99%) and Tin(II) 2-ethylhexanoate ($Sn(Oct)_2$) were purchased from Aldrich and used as received. TDI (toluene-2,4-diisocyanate, 80% of 2,4-isomer) was purchased from Aldrich, distilled in vacuum and freeze out to separate the 2,6-isomer. FOH (furfuryl alcohol, 98%) was purchased from Aldrich and distilled under reduced pressure to remove tarry material. FA (furfurylamine, 99%) was purchased from Acros, dried over NaOH and distilled in vacuum prior to use. PPG2000 (polypropylene glycol, Mn = 2000) was purchased from Aldrich and dried under vacuum at 110 °C prior to use. DMF (N,N-dimethylformamide) was purchased from Acros dried over CaH_2 and distilled in vacuum prior to use. Methylene chloride (CH_2Cl_2) was purchased from Acros, dried over P_2O_5 and distilled.

2. Synthesis and Sample Preparation

2.1 Synthesis of DFA-T

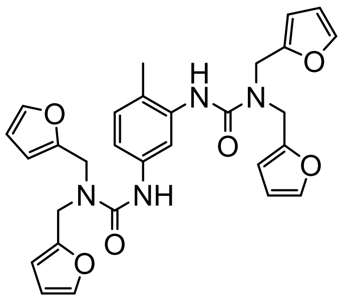

Synthesis was performed in argon atmosphere. DFA (2.0 g, 11.5 mmol) was added portionwise to the cooled in ice bath TDI (1.0 g, 5.8 mmol). Reaction mixture solidified in 10 minutes after reagents mixing. The product was purified by reprecipitation from CH_2Cl_2/hexane and dried in air given 2.5 g (81%) of colorless amorphous powder. ^{1}H NMR (600 MHz, DMSO-*d₆*) δ/ppm: 2.05 (s, 3H), 4.53 – 4.55 (m, 8H), 6.32 (dd, J = 10.6, 3.1 Hz, 4H), 6.41 (ddd, J = 11.8, 3.1, 1.8 Hz, 4H), 7.03 (d, J = 8.4 Hz, 1H), 7.23 (dd, J = 8.2, 2.2 Hz, 1H), 7.45 (d, J = 2.2 Hz, 1H), 7.62 (ddd, J = 11.0, 1.7, 0.7 Hz, 4H), 8.03 (s, 1H), 8.55 (s, 1H). ^{13}C NMR (101 MHz, DMSO-d_6) δ/ppm: 17.57, 42.71, 108.43, 110.91, 116.86, 118.03, 126.74, 129.98, 137.87, 138.50, 143.04, 151.76, 155.10, 155.44. IR (ATR, neat, cm^{-1}): 731, 808, 864, 884, 1011, 1070, 1148, 1225, 1259, 1520, 1506, 1602, 1634, 3305.

2.2 Synthesis of FA-T

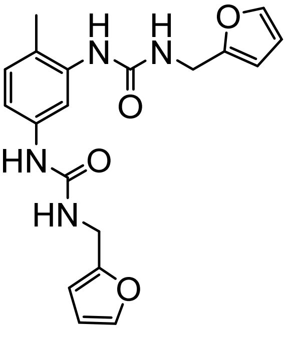

FA-T was obtained the same method as DFA-T from FA (1.4 g, 14.1 mmol) and TDI (1.2 g, 7.1 mmol) as slightly yellow solid, 1.9 g (73%). ^{1}H NMR (600 MHz, DMSO-d_6)

δ/ppm: 2.09 (s, 3H), 4.25 – 4.32 (m, 4H), 6.24 – 6.29 (m, 2H), 6.37 – 6.42 (m, 3H), 6.94 – 6.97 (m, 2H), 7.15 (dd, J = 8.2, 2.2 Hz, 1H), 7.57 – 7.61(m, 2H), 7.63 (s, 1H), 7.77 – 7.79 (m, 1H), 8.43 (s, 1H).^{13}C NMR (101 MHz, DMSO-d_6) δ/ppm: 17.24, 36.21, 106.58, 110.12, 110.52, 111.79, 119.44, 130.08, 138.2, 142.14, 153.2, 155.02. IR (ATR, neat, cm^{-1}): 740, 814, 884, 916, 1011, 1078, 1148, 1241, 1270, 1506, 1561, 1602, 1631, 3305.

2.3 Synthesis of FOH-T

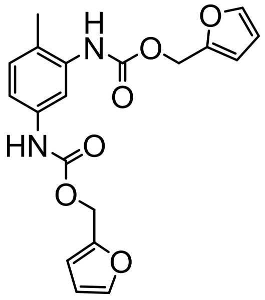

To FOH (2.3 g, 11.5 mmol) in 20 ml of DMF in argon atmosphere at 0 °C TDI (2.0 g, 23.0 mmol) was added. Reaction mixture was allowed to warm at room temperature and then stirred for 48 hrs at 60 °C. Reaction progress was monitored by IR by the disappearance of NCO band at 2265 cm^{-1}. The product was purified by reprecipitation from CH_2Cl_2/diethyl ether and dried in air given 2.8 g (66%) of colorless amorphous powder. ^{1}H NMR (600 MHz, DMSO-d_6) δ/ppm: 2.11 (s, 3H), 5.08 – 5.11 (m, 4H), 6.45 – 6.50 (m, 2H), 6.54 – 6.58 (m, 2H), 7.05 – 7.09 (m, 1H), 7.14 – 7.19 (m, 1H), 7.50 – 7.57 (m, 1H), 7.68 – 7.72 (m, 2H), 8.98 (s, 1H), 9.71 (s, 1H). ^{13}C NMR (101 MHz, DMSO-d_6) δ/ppm: 17.17, 57.76, 110.79, 130.39, 136.38, 137.07, 143.69,149.87, 153.05, 153.90. IR (ATR, neat, cm^{-1}): 738, 751, 809, 884, 916, 1016, 1062, 1148, 1241, 1265, 1506, 1520, 1689, 1716, 3252.

2.4 Synthesis of DFA-M

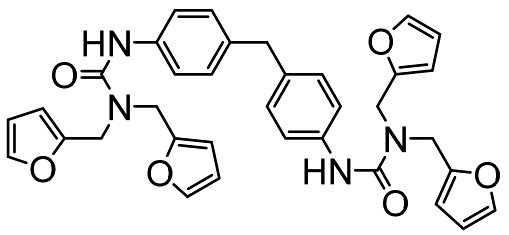

Synthesis was performed in argon atmosphere. DFA (2.8 g, 16.0 mmol) was added portionwise to the cooled in ice bath MDI (2.0 g, 8.0 mmol) in DMF (5 ml). Reaction mixture was stirred at room temperature for 20 minutes. Cold diethyl ether was added to the reaction mixture, colorless solid was filtered, additionally washed with ether and dried in air given 4.4 g (92%) of colorless amorphous powder. ^{1}H NMR (600 MHz, DMSO-d_6) δ/ppm 3.79 (s, 2H), 4.52 (s, 8H), 6.30 (d, J = 3.1 Hz, 4H), 6.40 (dd, J = 3.1, 1.9 Hz, 4H), 7.08 (d, J = 8.5 Hz, 4H), 7.38 (d, J = 8.5 Hz, 4H), 7.60 (dd, *J* = 1.7, 0.7 Hz, 4H), 8.53 (s, 2H). ^{13}C NMR (101 MHz, DMSO-d_6) δ/ppm: 42.67, 108.54, 110.94, 120.51, 128.96, 135.74, 138.55, 143.10, 151.68, 155.20. IR (ATR, neat, cm^{-1}): 743, 758, 774, 813, 884, 1011, 1070, 1146, 1211, 1252, 1303, 1414, 1476, 1513, 1520, 1593, 1635, 3267.

2.5 Synthesis of FA-M

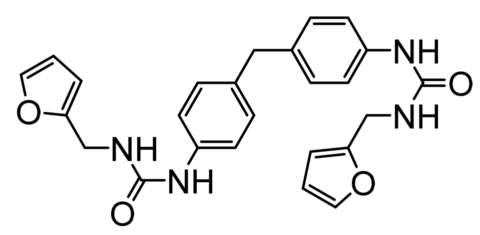

FA-M was obtained the same method as DFA-M from FA (1.6 g, 16.0 mmol) and MDI (2.0 g, 8.0 mmol) as slightly yellow solid, 3.2 g (90%). ^{1}H NMR (600 MHz, DMSO-*d$_6$*) δ/ppm 3.76 (s, 3H), 4.28 (d, *J* = 8.2 Hz, 2H), 6.25 (d, J = 3.2 Hz, 2H), 6.39 (dd, J = 3.2, 1.8 Hz, 2H), 6.50 (t, J = 5.8 Hz, 2H), 7.05 (d, J = 8.2 Hz, 4H), 7.33 – 7.27 (m, 4H), 7.58 (dd, *J* = 1.9, 0.9 Hz, 2H), 8.45 (s, 2H). ^{13}C NMR (101 MHz, DMSO-*d$_6$*) δ/ppm: 26.51, 30.25, 40.61, 42.26, 108.15, 110.83, 142.83, 152.08, 157.19. ^{13}C NMR (151 MHz, DMSO) δ 155.42, 153.61, 142.48, 138.64, 134.93, 129.27, 118.36, 110.92, 106.93, 36.57. IR (ATR, neat, cm^{-1}): 731, 744, 774, 779, 813, 884, 913, 1011, 1150, 1239, 1303, 1409, 1510, 1569, 1593, 1635, 3305.

2.6 Synthesis of FOH-M

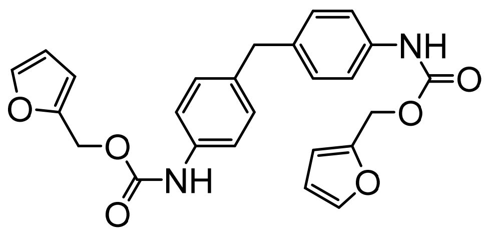

FOH-M was obtained the same method as DFA-T from FOH (1.6 g, 16.0 mmol) and MDI (2.0 g, 8.0 mmol) as slightly yellow solid, 3.5 g (96%). ^{1}H NMR (600 MHz, DMSO-*d$_6$*) δ/ppm 3.78 (s, 2H), 5.08 (s, 4H), 6.46 (dd, *J* = 3.2, 1.9 Hz, 2H), 6.55 (d, J = 3.2 Hz, 2H), 7.09 (d, J = 8.5 Hz, 4H), 7.34 (d, J = 8.1 Hz, 4H), 7.69 (dd, J = 1.8, 0.8 Hz, 2H), 9.69 (s, 2H). ^{13}C NMR (101 MHz, DMSO-*d$_6$*) δ/ppm: 58.08, 111.23, 118.78, 129.37, 136.10, 137.33, 144.13, 150.18, 153.48. IR (ATR, neat, cm^{-1}): 741, 774, 793, 885, 916, 1015, 1065, 1152, 1239, 1305, 1415, 1533, 1593, 1702, 3307.

2.7 Synthesis of DFA-H

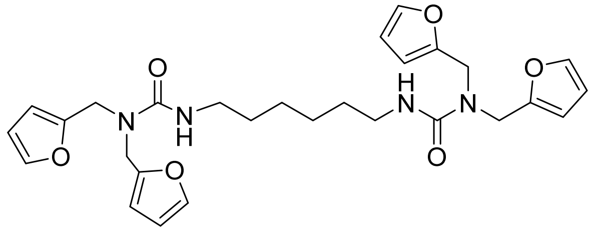

DFA (60 mmol) in 1 ml DMF was added to HDI (30 mmol) in argon, then 0,08 mmol of Sn(Oct)$_2$ was added in the reaction mixture. The reaction was stirred 8 h at room temperature and controlled by IR spectroscopy: the diisocyanate band at 2217 cm^{-1} disappeared. The resulting adduct was purified by recrystallization from dichloromethane/petroleum ether and washed with diethyl ether. Adduct - white solid (6 g, 40 %). ^{1}H NMR (400 MHz, DMSO-*d*$_6$) δ/ppm 1.22 – 1.13 (m, 4H), 1.39 – 1.29 (m, 9H), 3.01 (q, *J* = 6.5 Hz, 4H), 4.34 (s, 8H), 6.20 (d, *J* = 3.2 Hz, 4H), 6.35 (dd, *J* = 3.2, 1.8 Hz, 4H), 6.51 (t, *J* = 5.5 Hz, 2H), 7.54 (d, *J* = 1.8 Hz, 4H). ^{13}C NMR (101 MHz, DMSO-*d*$_6$) δ/ppm: 26.51, 30.25, 40.61, 42.26, 108.15, 110.83, 142.83, 152.08, 157.19. IR (ATR, neat, cm^{-1}): 737, 1013, 1077, 1150, 1222, 1268, 1343, 1541, 1627, 2937, 3351.

2.8 Synthesis of FA-H

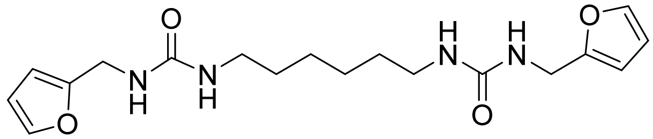

1,1'-(hexane-1,6-diyl)bis(3-(furan-2-ylmethyl)urea) was prepared by modified method [3]. HDI (6 mmol) and FA (6 mmol) in 5 ml of DCM (dichloromethane) and 0,1 mmol of $Sn(Oct)_2$ was charged in two-necked flask with argon at 0 °C. Reaction mass was stirred at room temperature overnight. The resulting symmetric diurea was purified by recrystallization from dichloromethane / petroleum ether and washed with diethyl ether. Adduct - white solid (4, 42 g, 85 %). ^{1}H-NMR (600 MHz, d6-DMSO) δ/ppm: 1.23(s, 4H), 1.34(s, 4H), 2.09(s, 2H), 2.96(m, 4H), 4.17(s, 4H), 5.90(s, 2H), 6.17(s, 2H), 6.37(s, 2H), 7.55(s, 2H). ^{13}C-NMR (DMSO-d6, ppm. J, Hz): 26.55, 30.41, 36.75, 39.64, 106.49, 110.79, 142.23, 154.21, 158.14. IR (ATR, neat, cm^{-1}): 741, 917, 1070. 1257, 1578, 1619, 2858, 2928, 3314.

2.9 Synthesis of FOH-H

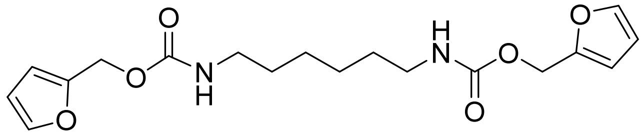

Bis(furan-2-ylmethyl) hexane-1,6-diyldicarbamate was prepared by modified method [3]. HDI (6 mmol) and FOH (6 mmol) in 5 ml of DCM (dichloromethane) and 0,1 mmol of $Sn(Oct)_2$ was charged in two – necked flask with argon at room temperature. The reaction mixture was refluxed for 2 hrs. The resulting adduct was purified by recrystallization from dichloromethane / petroleum ether and washed with diethyl ether. Adduct - white solid (1,7 g, 79 %). ^{1}H-NMR (600 MHz, d6-DMSO) δ/ppm: 1.25 – 1.19 (m, 4H), 1.39 – 1.33 (m, 4H), 2.96 (q, J = 7.4, 7.0 Hz, 5H), 4.95 (d, J = 7.9 Hz, 3H), 6.46 (d, J = 16.7 Hz, 4H), 7.27 – 7.22 (m, 2H), 7.68 – 7.63 (m, 2H). ^{13}C-NMR (DMSO-d6, ppm. J, Hz): 26.38, 29.79, 40.64, 57.67, 110.78, 111.21, 143.85, 150.74, 156.17. IR (ATR, neat, cm^{-1}): 745, 921, 1005, 1257, 1343, 1537, 1693, 2953, 3327.

2.10 Synthesis of linear polyurethane PU-HEMI

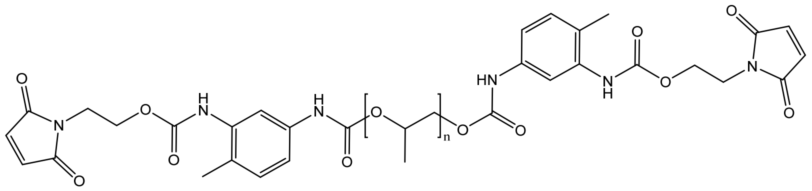

Linear PU prepolymer was prepared via conventional two-step method. At the first step TDI (3.66 g, 1 eq) reacted with PPG2000 (21.07 g, 2 eq). The reaction was carried out at 60 °C under an argon atmosphere for 1.5 h. The reaction progress was monitored with IR-spectroscopy by decreasing the NCO-group band at 2271 cm^{-1}. At the second step reaction mixture was cooled to room temperature, diluted with DMF (10 mL) and HEMI (2.97 g, 2 eq) dissolved in DMF (10 mL) was added into the isocyanate end-capped

prepolymer solution. Reaction mixture was stirred at 60 °C under an argon atmosphere for 1.5 h till NCO-group band disappearance.

^1H NMR (400 MHz, DMSO-d_6, δ): 9.53 (s, 2H, -NH-), 8.81 (s, 2H, -NH-), 7.57 – 7.48 (m, 2H, Ar), 7.42 (s, 2H, Ar), 7.16 (s, 2H, Ar), 7.07 – 7.01 (m, 4H, -C=C- of maleimide), 4.89 – 4.81 (m, 2H, -(CH_3)C-**CH**-O), 4.22 – 4.17 (m, 4H,-**CH_2**-N-), 3.71 – 3.63 (m, 4H, -O-**CH_2**-), 3.55 – 3.38 (s, 63H, -(CH_3)C-**CH**-O), 3.35 – 3.25 (m, 32H, O-**CH**(CH_3)-), 2.20 – 1.95 (m, 6H, **CH_3**-Ar), 1.22 – 1.16 (m, 6H, **CH_3**-Ar), 1.10 – 0.97 (m, 96H, **CH_3**-CH2-); ^{13}C NMR (151 MHz, DMSO-d_6) δ 75.04, 72.91, 72.71, 17.71.; IR (ATR, neat, cm^{-1}): 3300, 2970, 2932, 2899, 2870, 1715, 1600, 1533, 1451, 1405, 1373, 1226, 1101, 1014, 927, 866, 829, 769, 696.

2.11 Synthesis of PU-DA.

FA-T (1.74 g, 3.8 mmol) in 70 mL of DMF was added to HEMI-prepolymer solution in DMF (10.0 g, 3.8 mmol, 0.35 g/ml). The reaction mixture was heated to 60 °C for 2 h, then carried onto glass surface and cured in an oven for 48 h to afford transparent yellow polymer film.

2.12 Sample preparation of PU-DA

Polymer solution in DMF (1.3 g mL^{-1}) was heated to 60 °C, poured into Petri dish, and kept in an oven at 60 °C for 48 h.

2.13 Self-healing ability test of PU-DA

Polymer sample was damaged by surgical blade (0.5 mm thickness) and subsequently undergone to the two-step healing cycle – 2h at 120 °C and 48 h at 60 °C.

3. Characterization

3.1 Nuclear Magnetic Resonance (NMR)

NMR spectra were recorded by a Bruker Avance 600 NMR Spectrometer (600.1 MHz), using residual proton signal of deuterated solvent as a reference, chemical shifts were reported as parts per million downfield from tetramethylsilane (TMS).

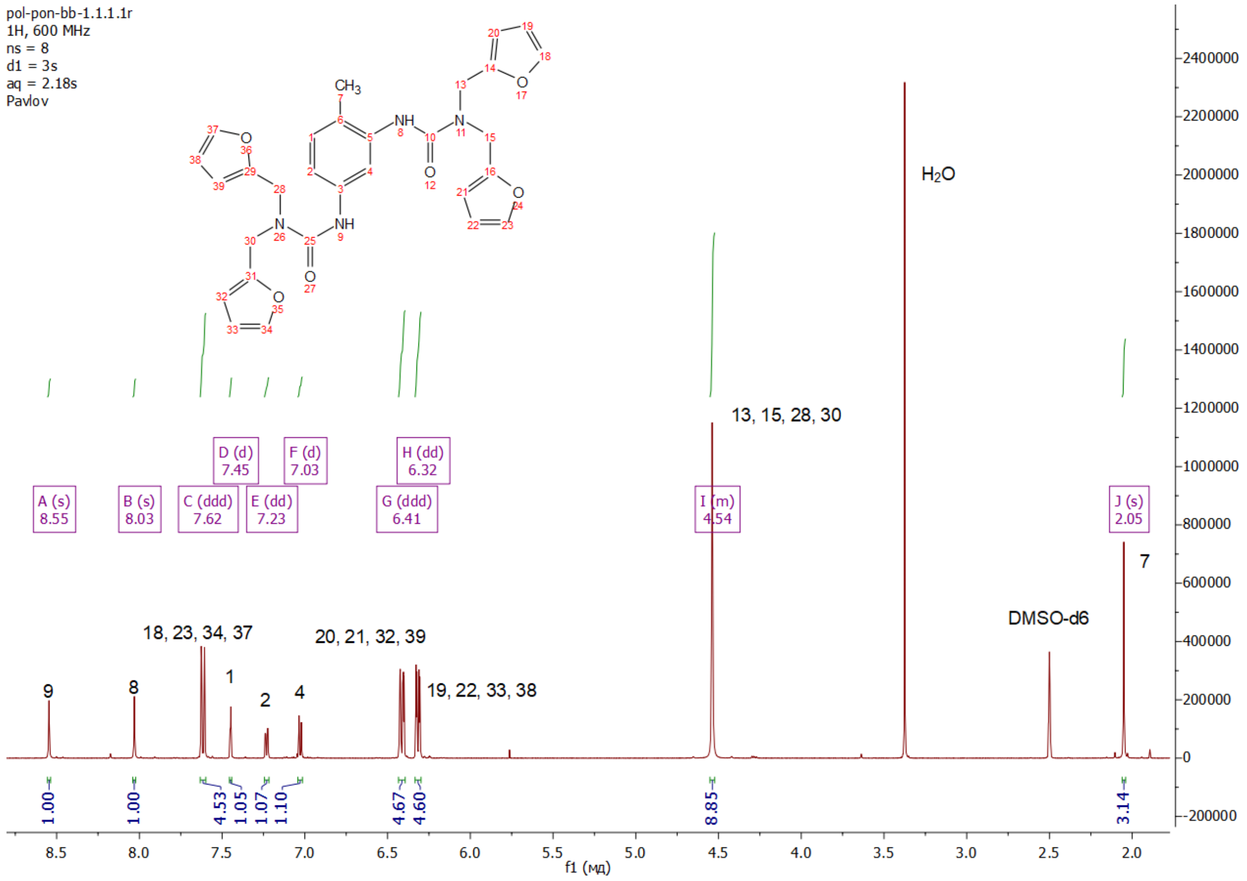

Figure S1 ^{1}H NMR spectrum of DFA-T in DMSO-d^{6}.

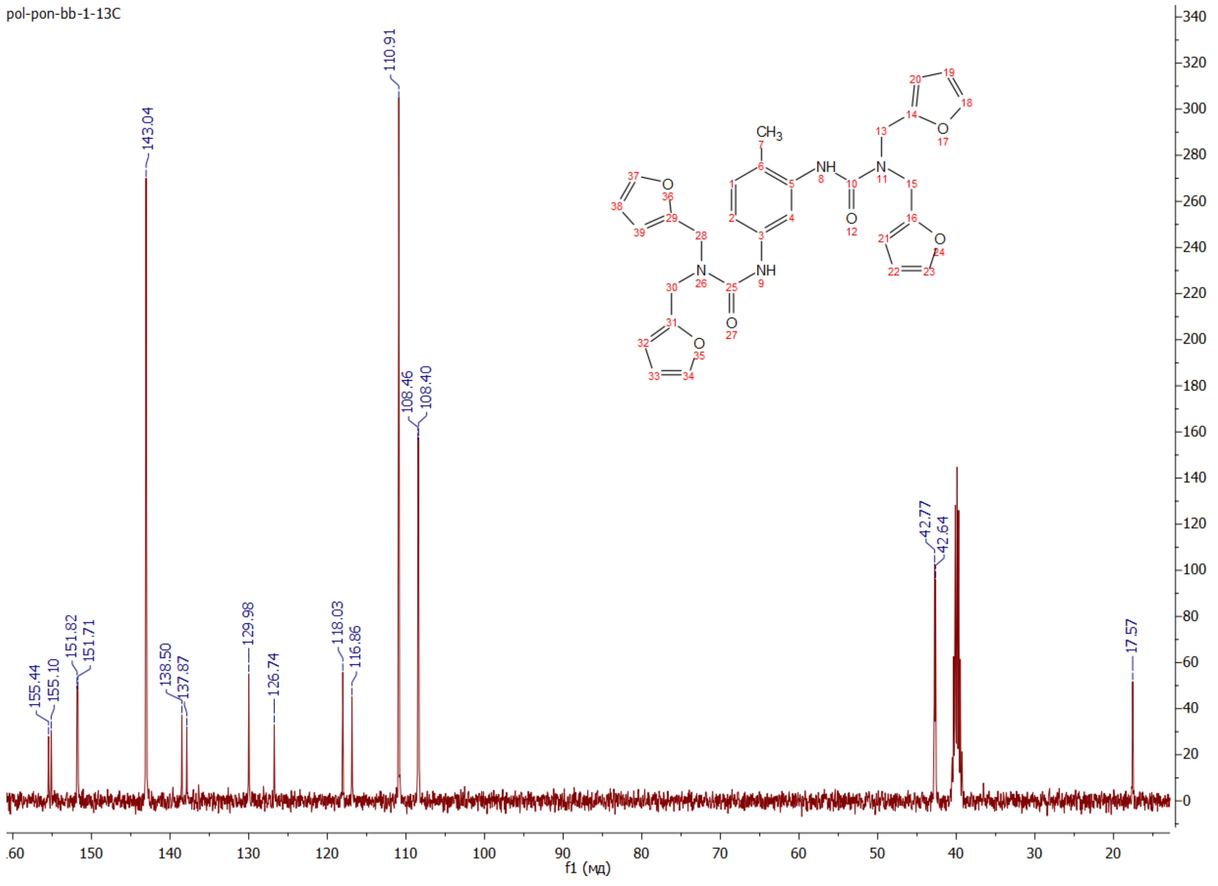

Figure S2 ^{13}C NMR spectrum of DFA-T in DMSO-d^{6}.

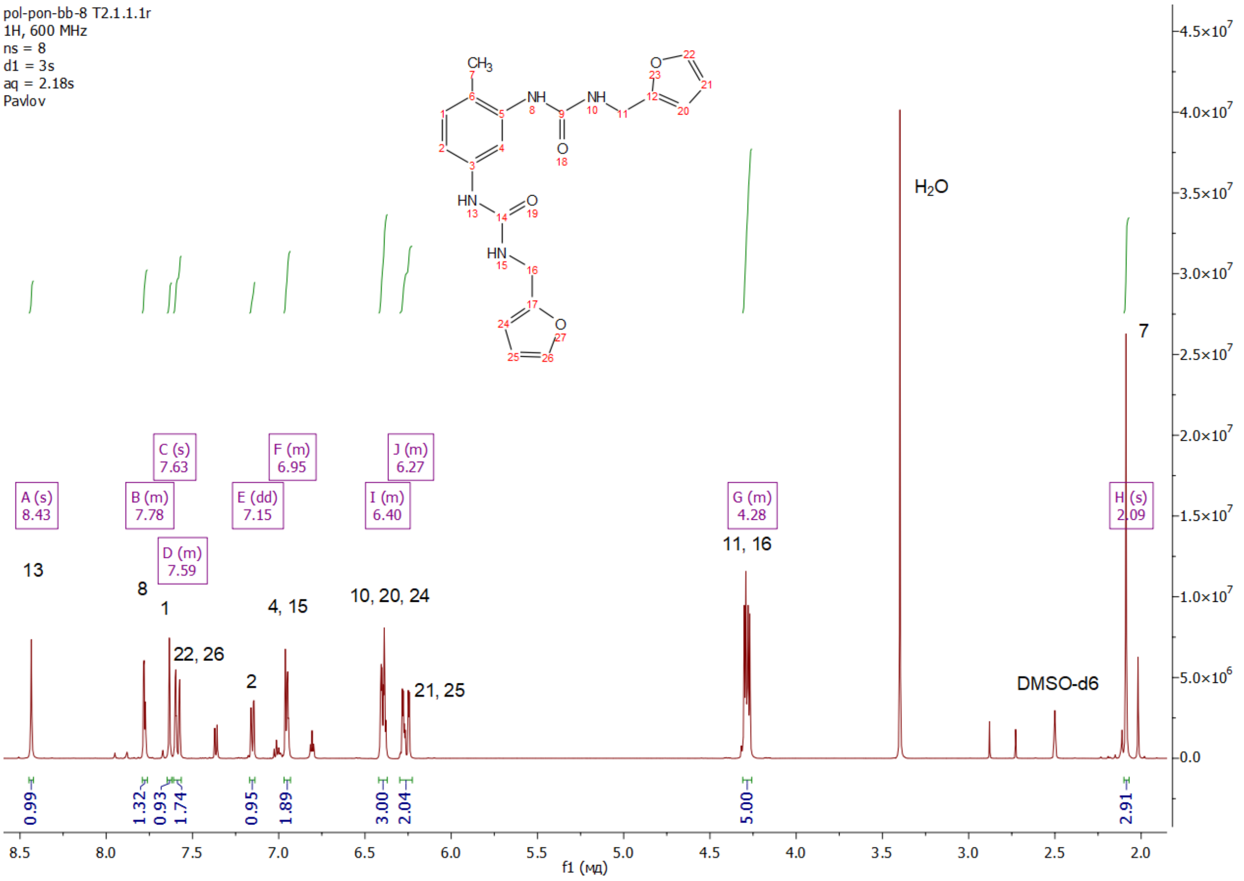

Figure S3 ^{1}H NMR spectrum of FA-T in DMSO-d^{6}.

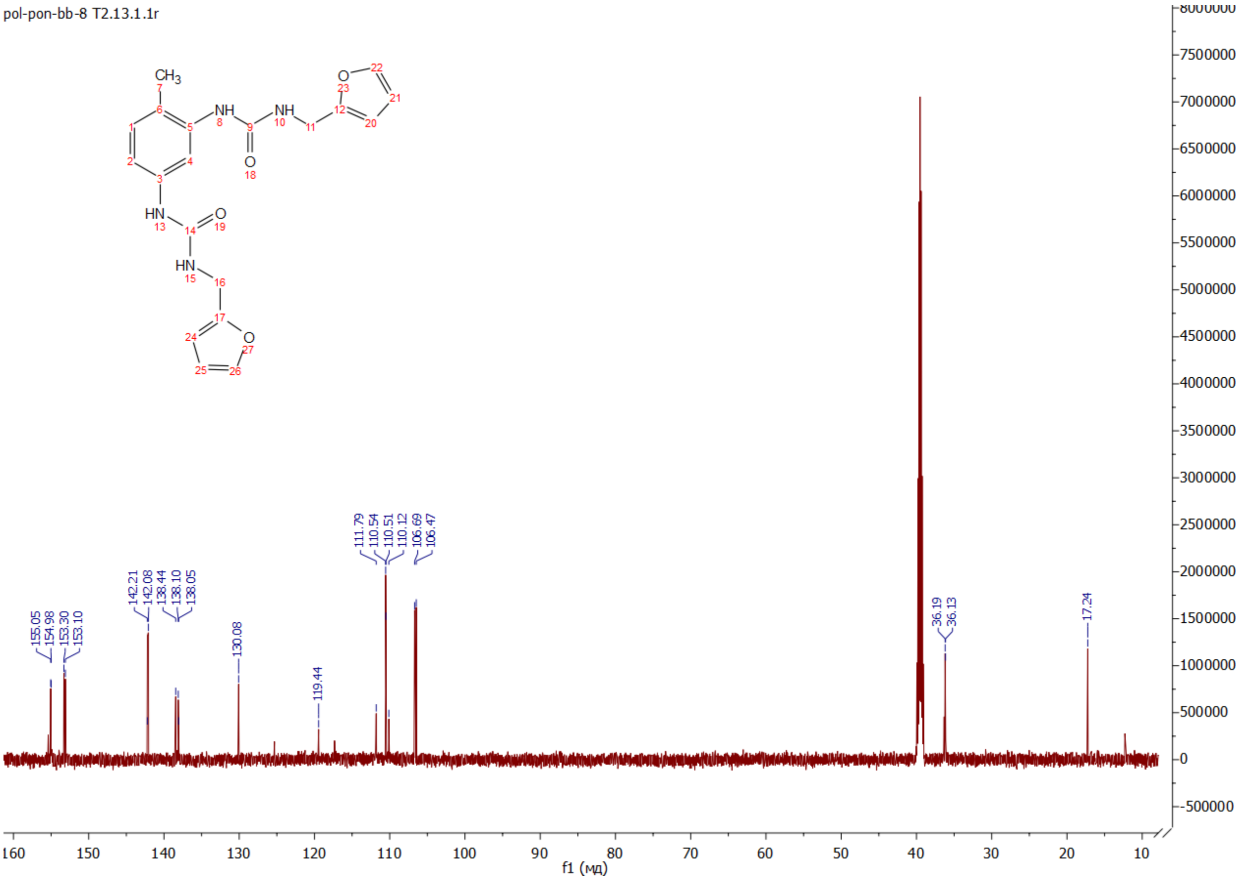

Figure S4 ^{13}C NMR spectrum of FA-T in DMSO-d^{6}.

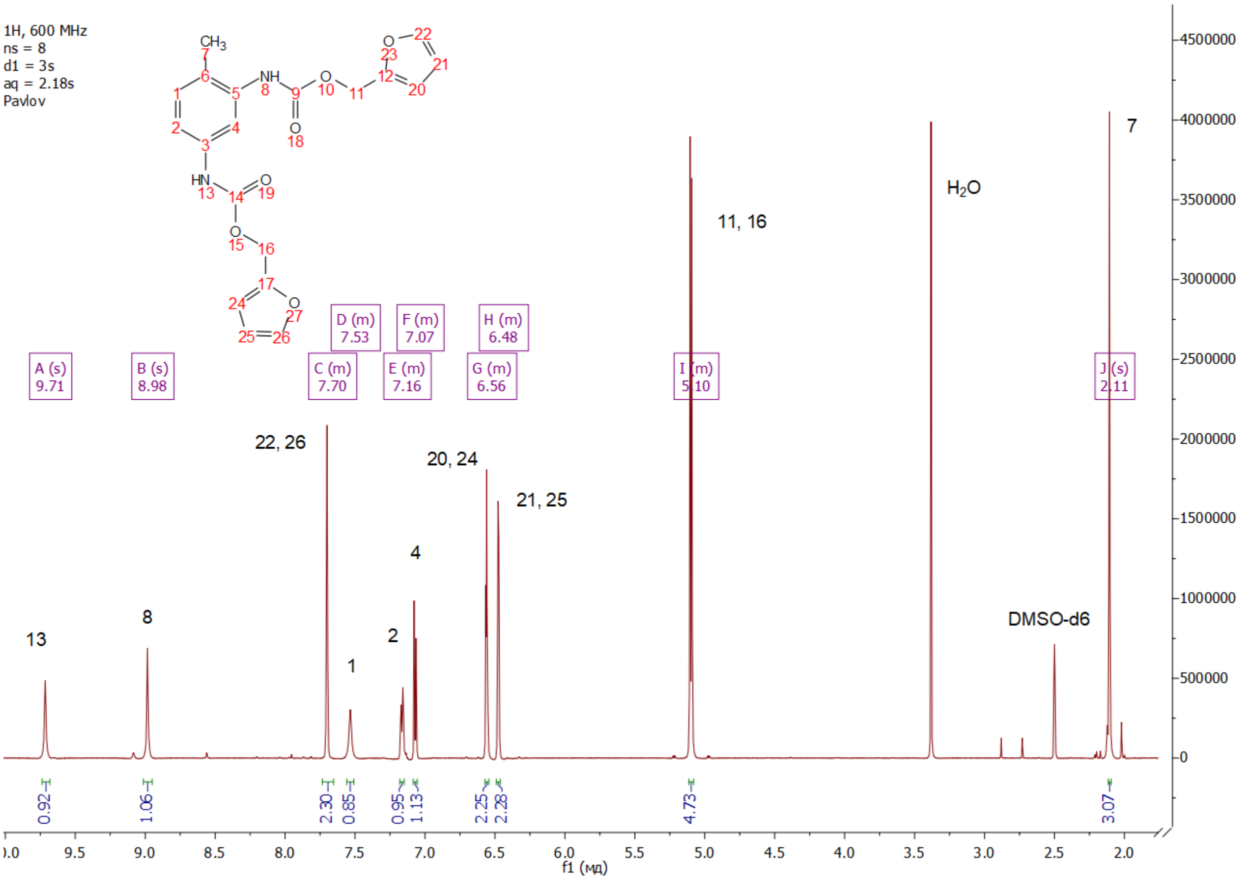

Figure S5 ^{1}H NMR spectrum of FOH-T in DMSO-d^{6}.

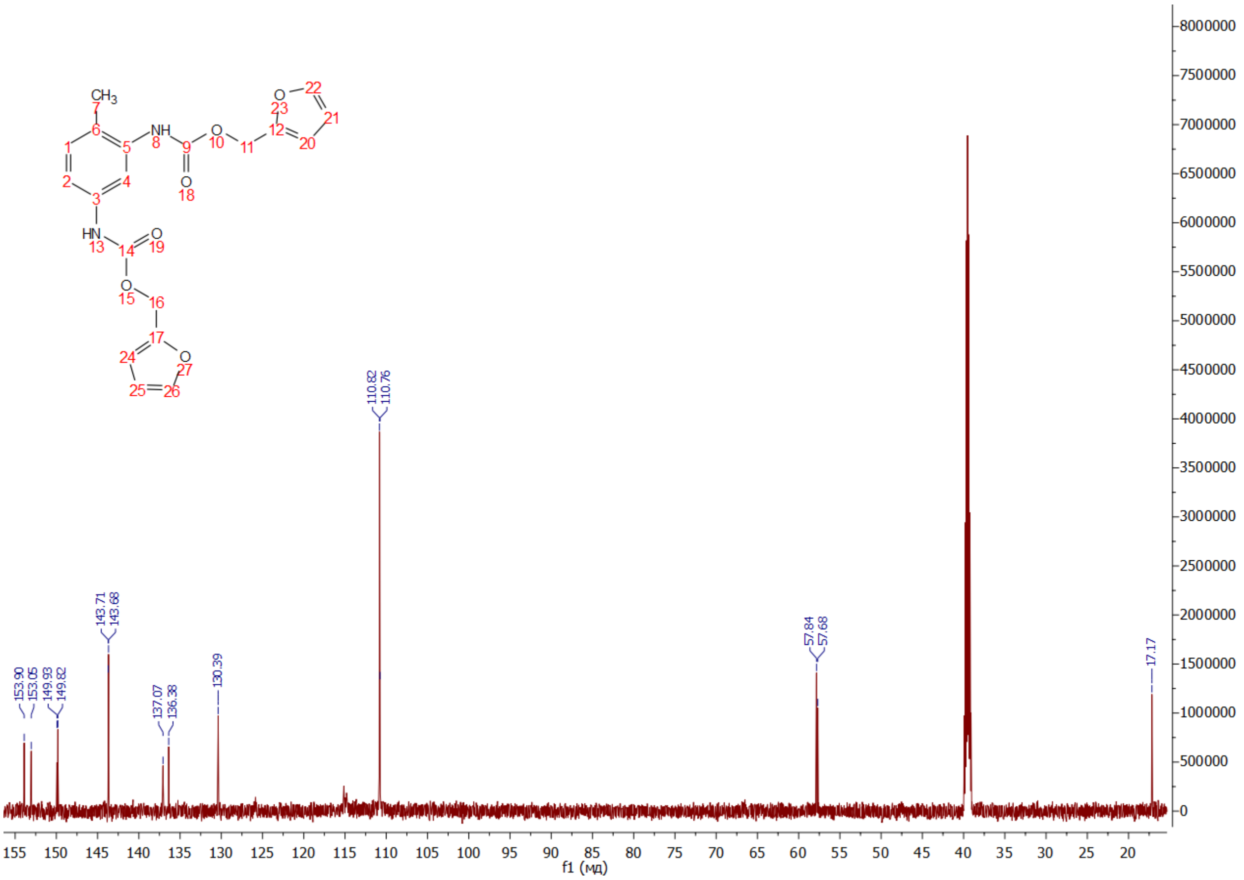

Figure S6 ^{13}C NMR spectrum of FOH-T in DMSO-d^{6}.

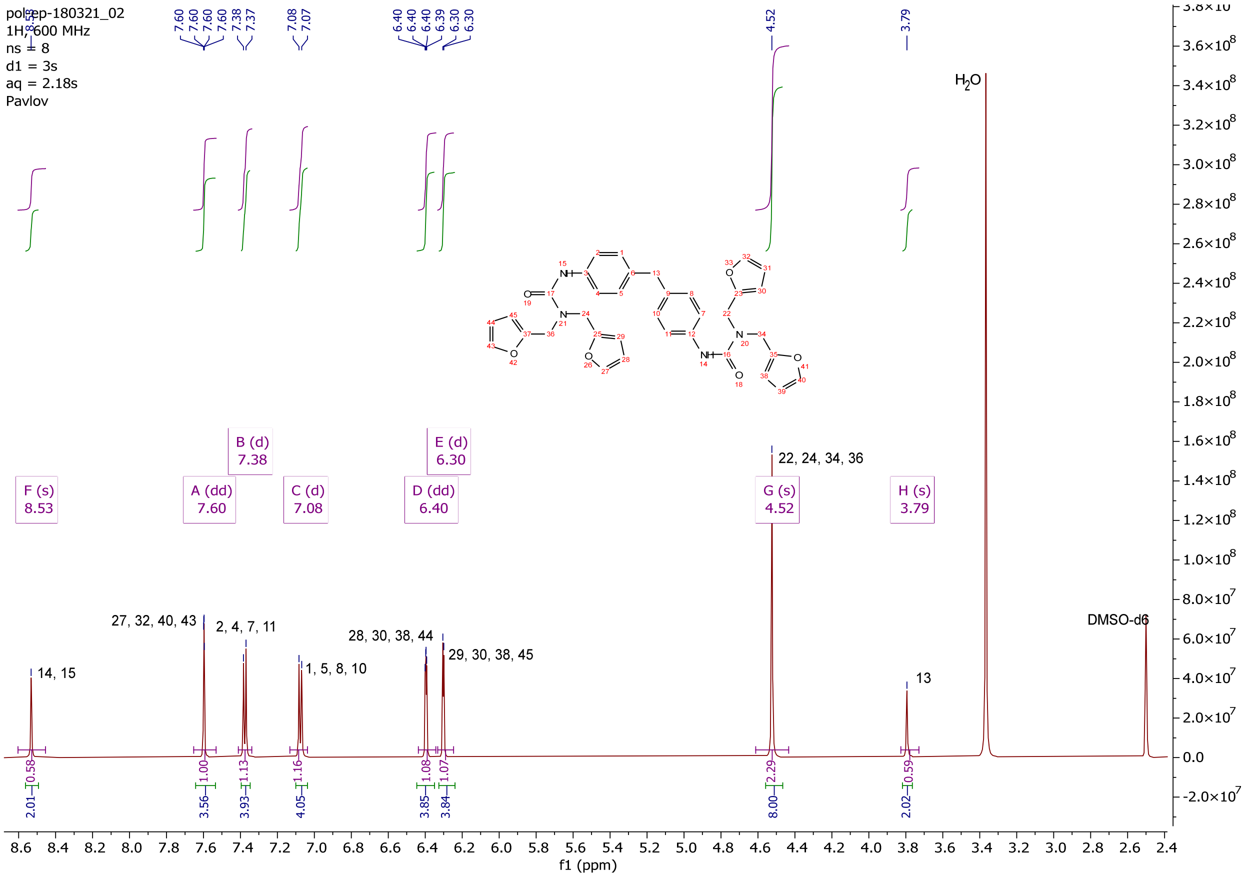

Figure S7 ^{1}H NMR spectrum of DFA-M in DMSO-d[6].

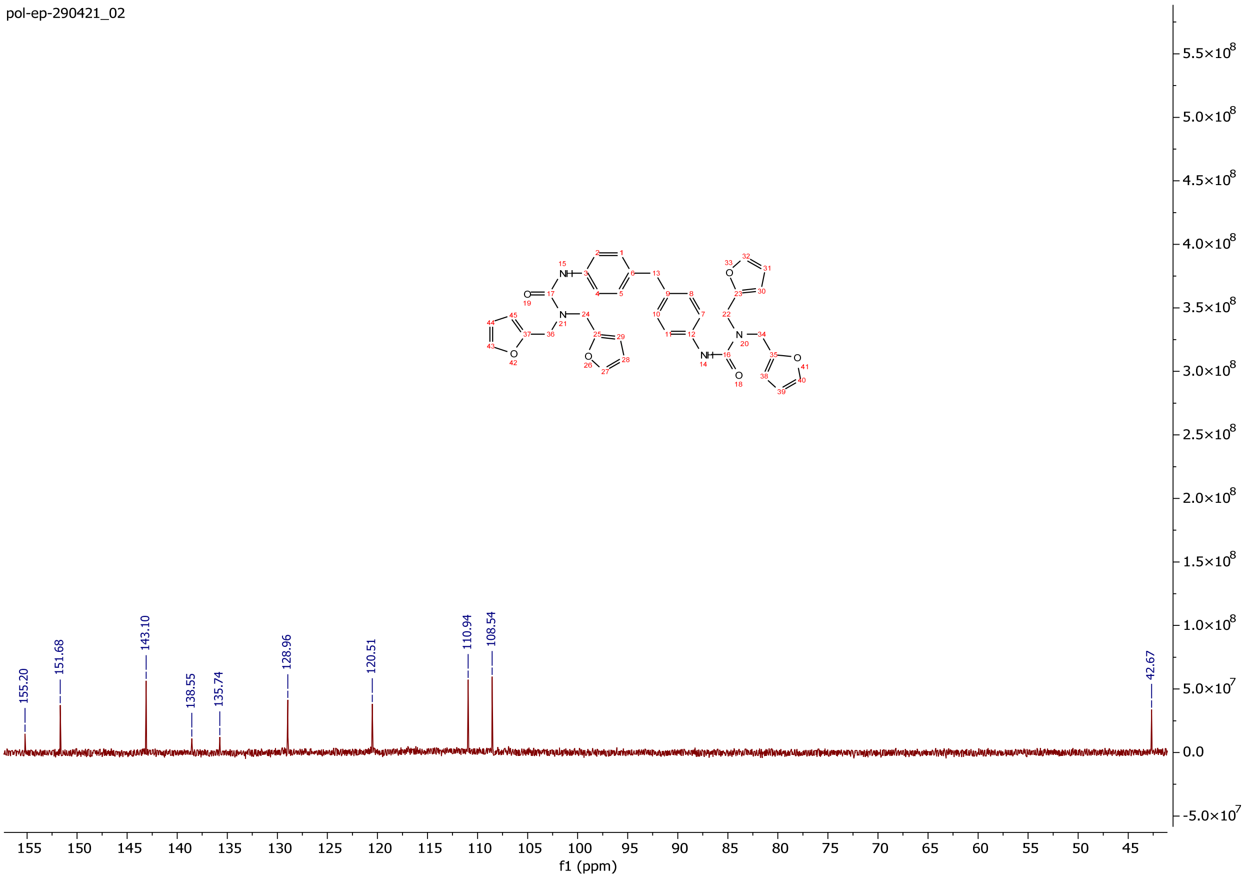

Figure S8 ^{13}C NMR spectrum of DFA-M in DMSO-d[6].

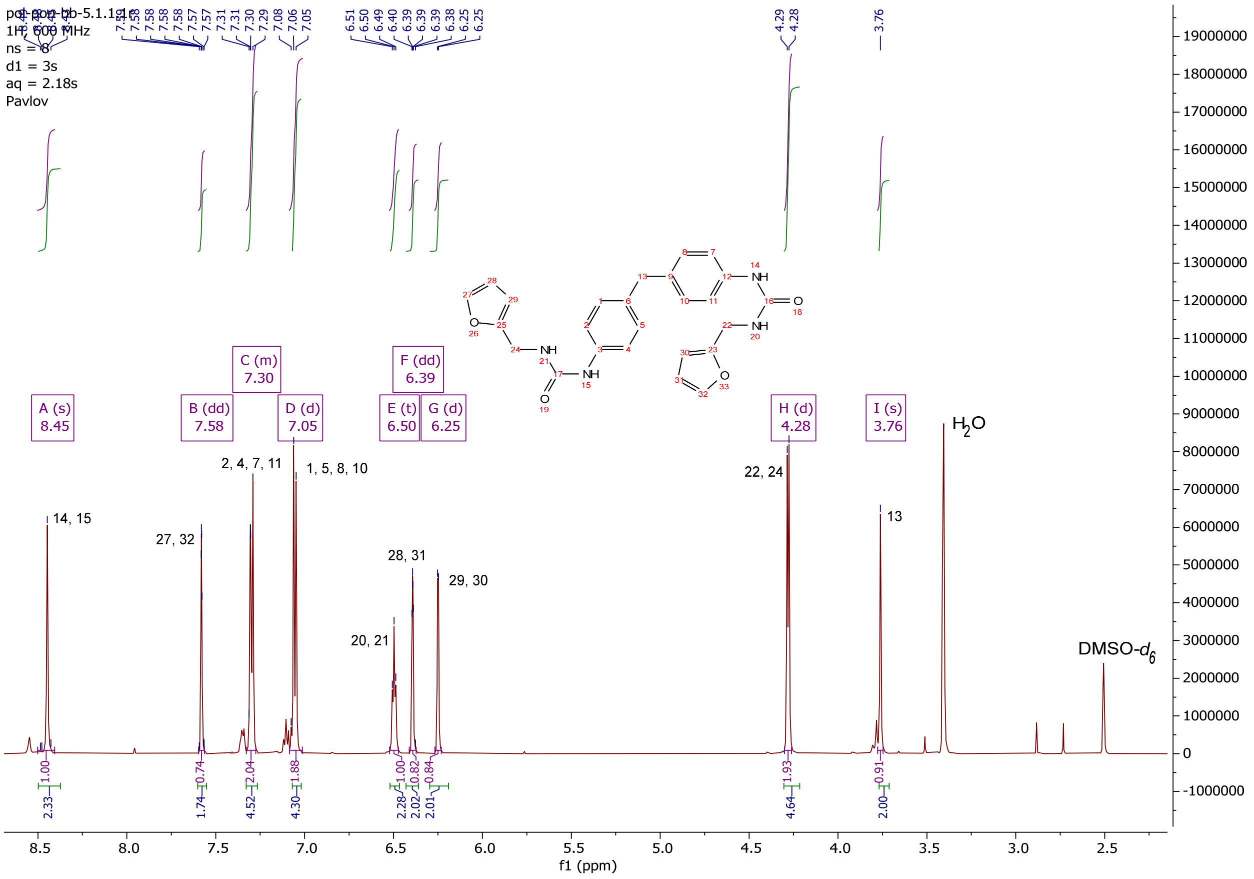

Figure S9 ^{1}H NMR spectrum of FA-M in DMSO-d_6.

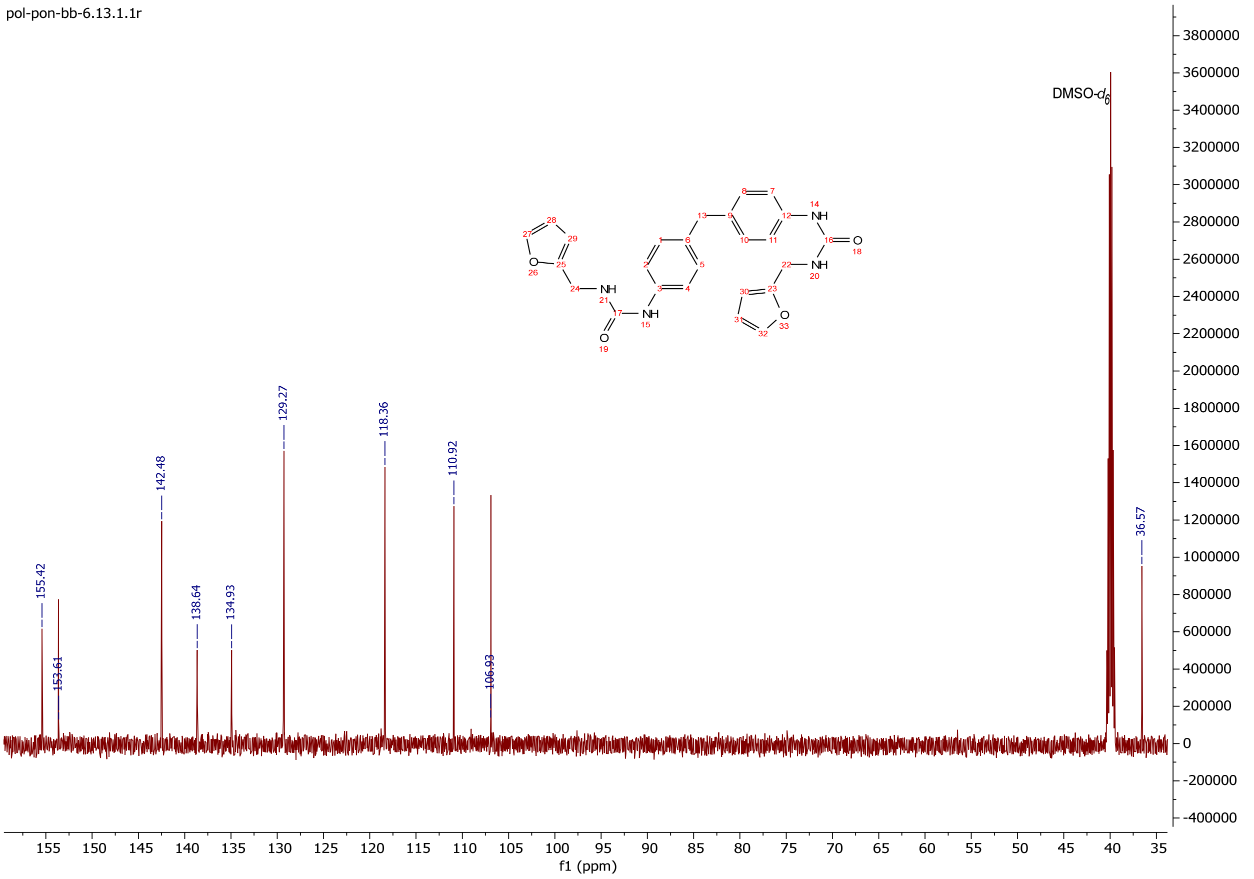

Figure S10 ^{13}C NMR spectrum of FA-M in DMSO-d_6.

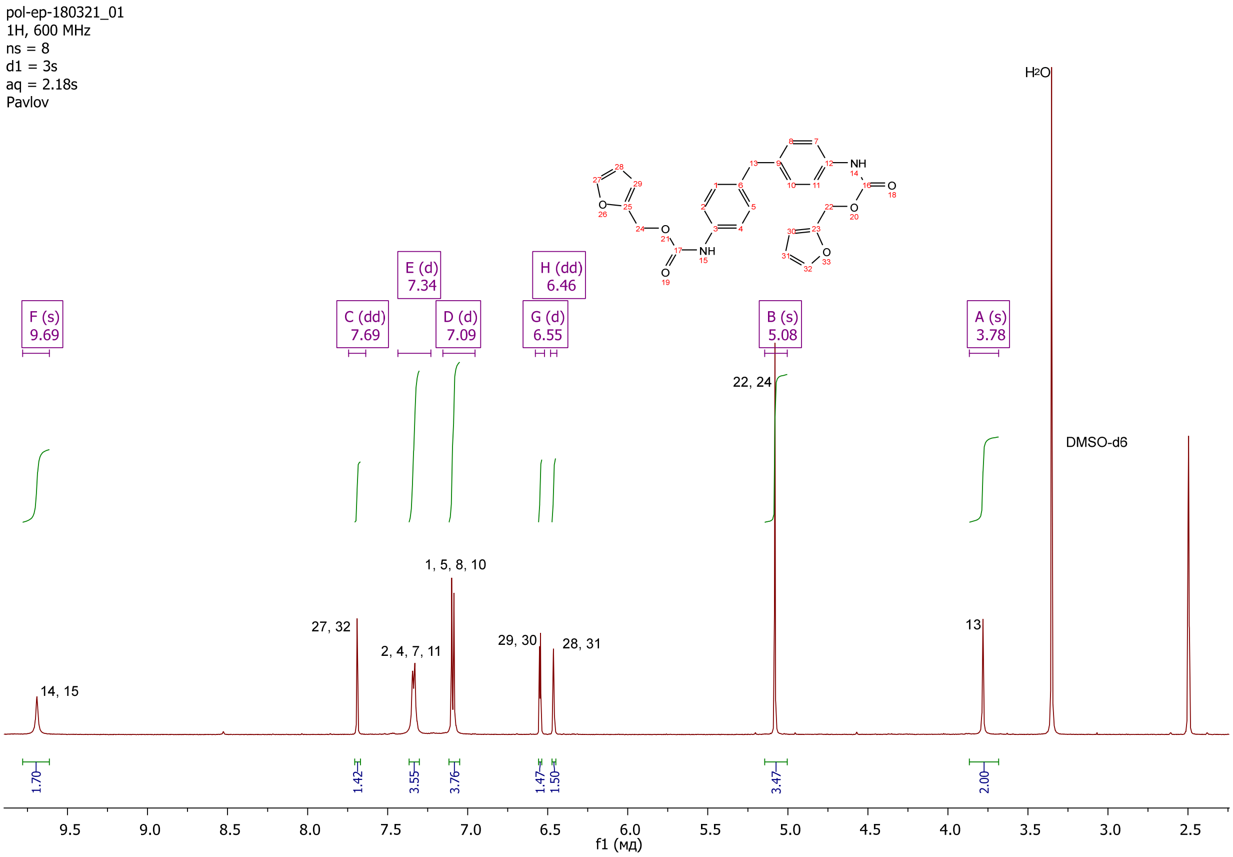

Figure S11 ^{1}H NMR spectrum of FOH-M in DMSO-d^{6}.

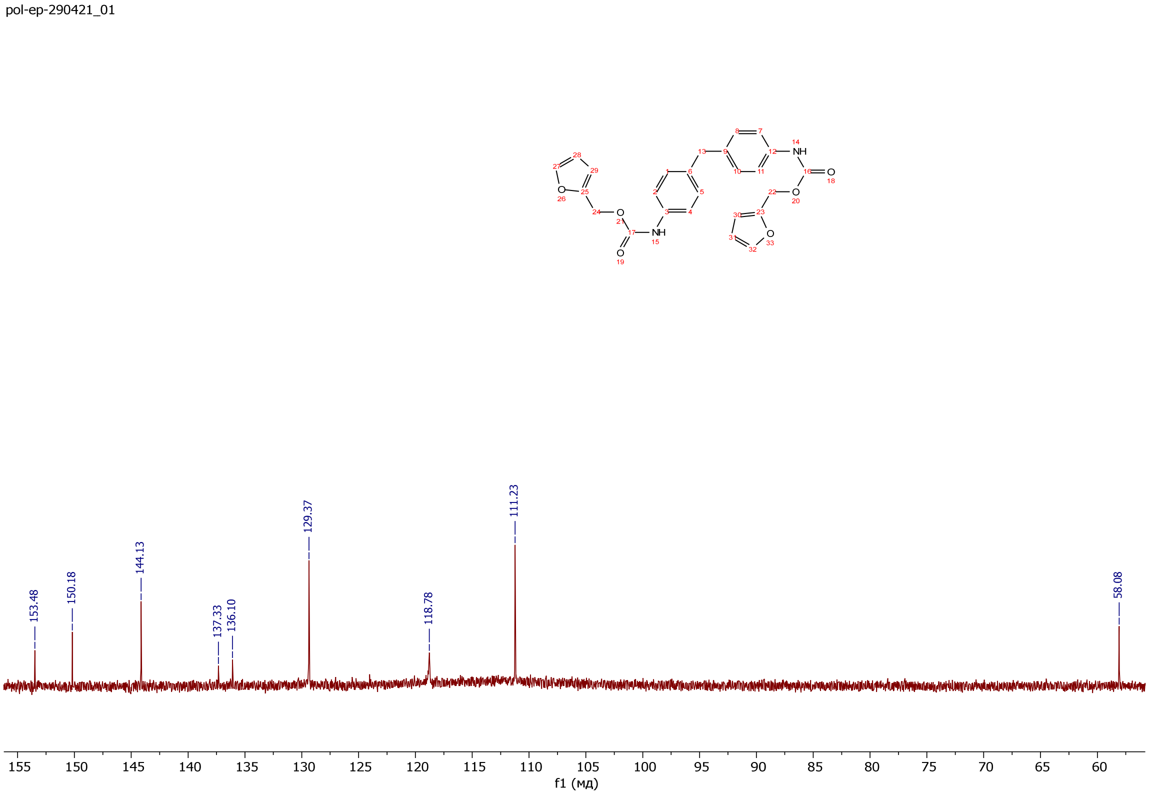

Figure S12 ^{13}C NMR spectrum of FOH-M in DMSO-d$_6$.

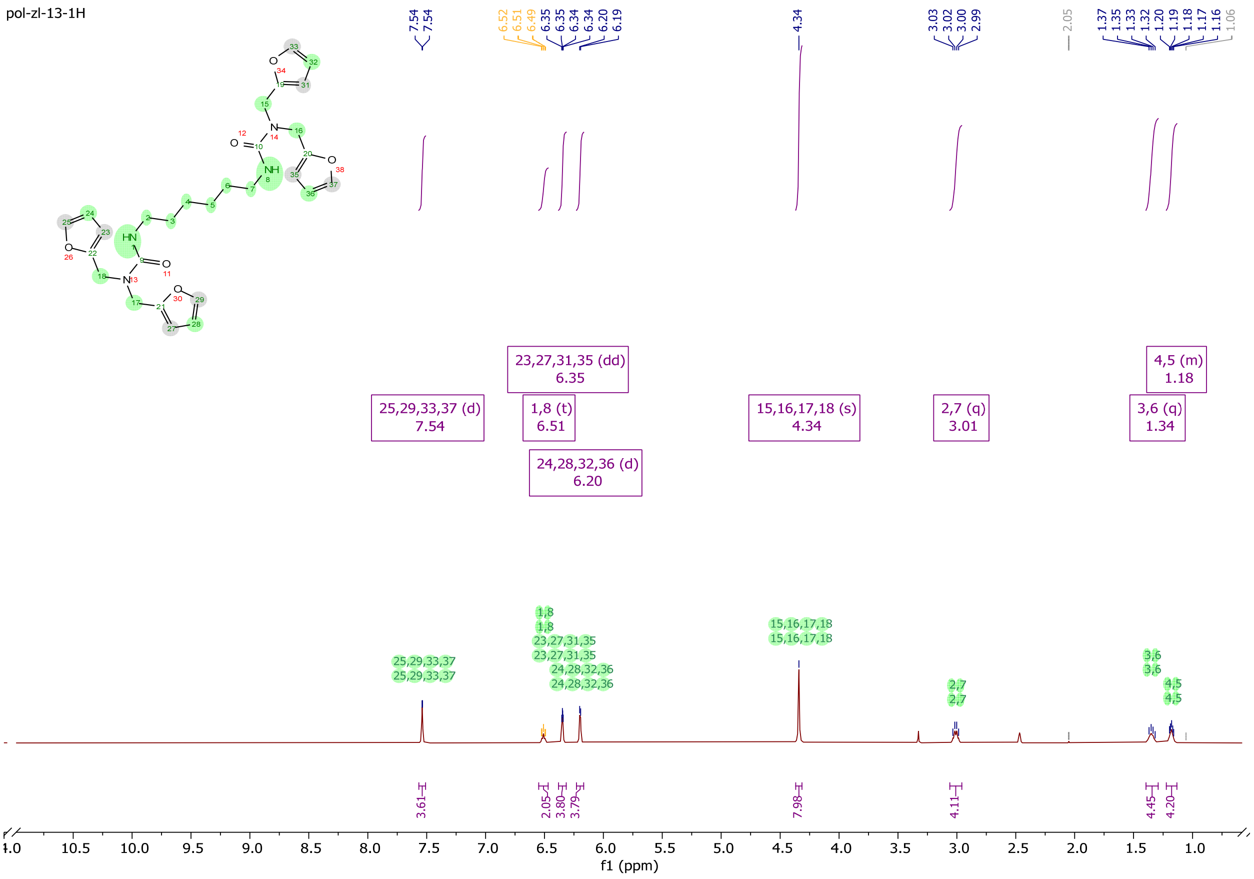

Figure S13 ^{1}H NMR spectrum of DFA-H in DMSO-d^{6}.

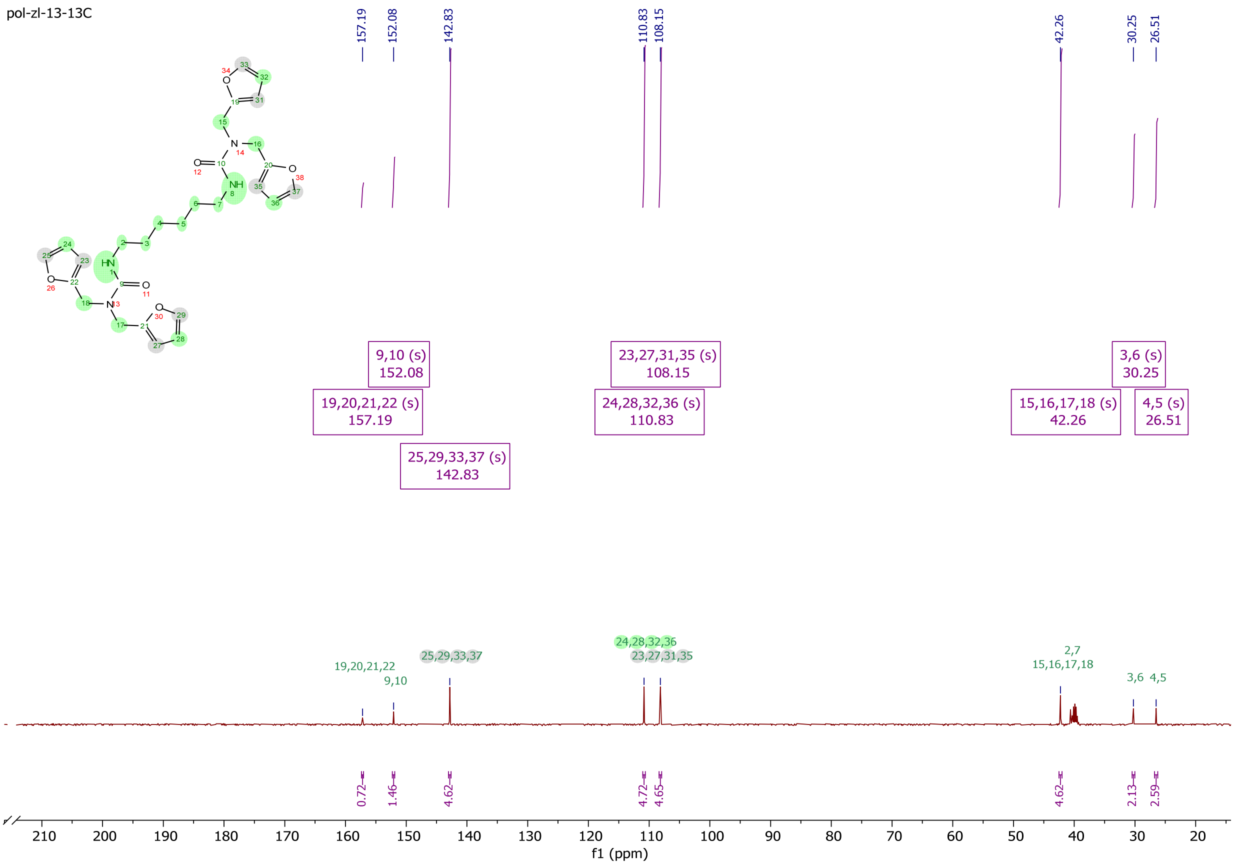

Figure S14 ^{13}C NMR spectrum of DFA-H in DMSO-d_6.

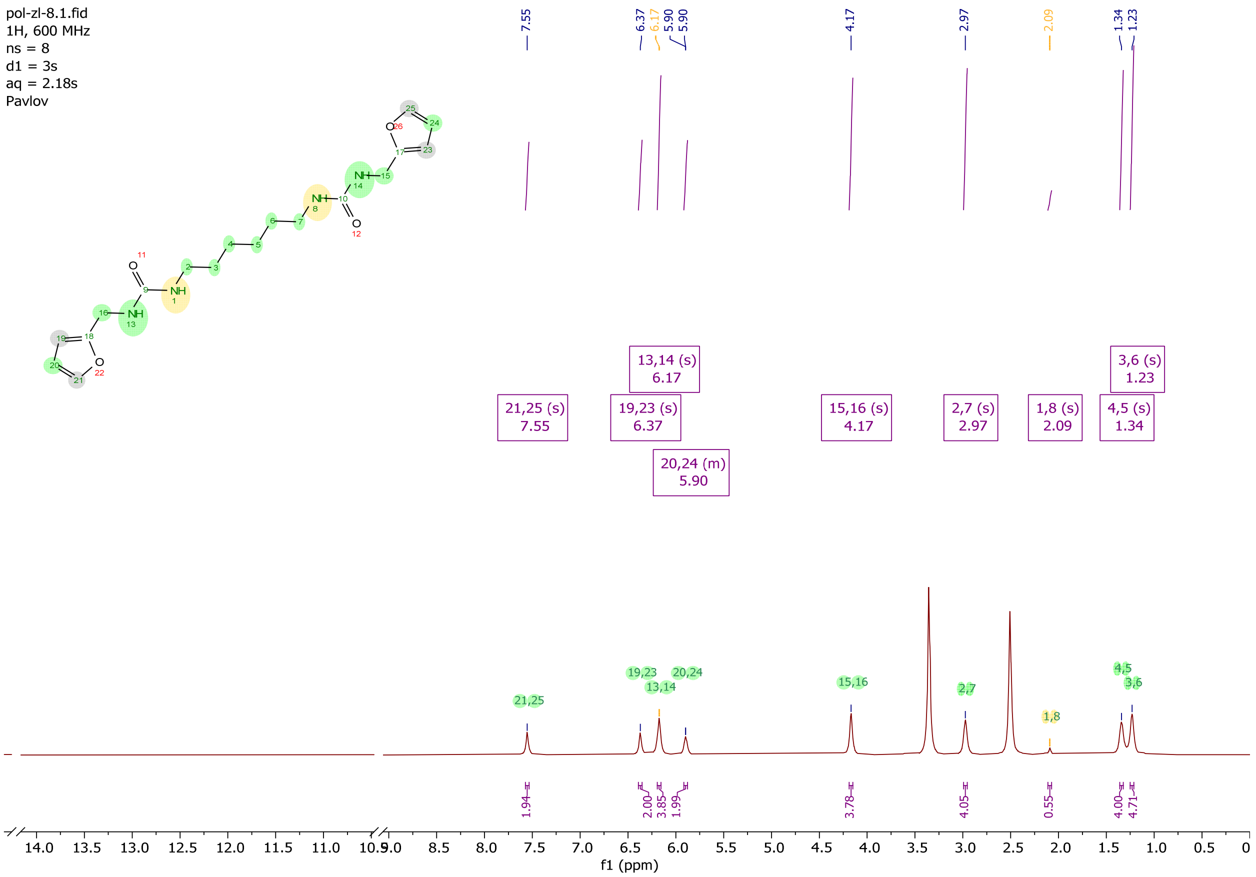

Figure S15 ^{1}H NMR spectrum of FA-H in DMSO-d^{6}.

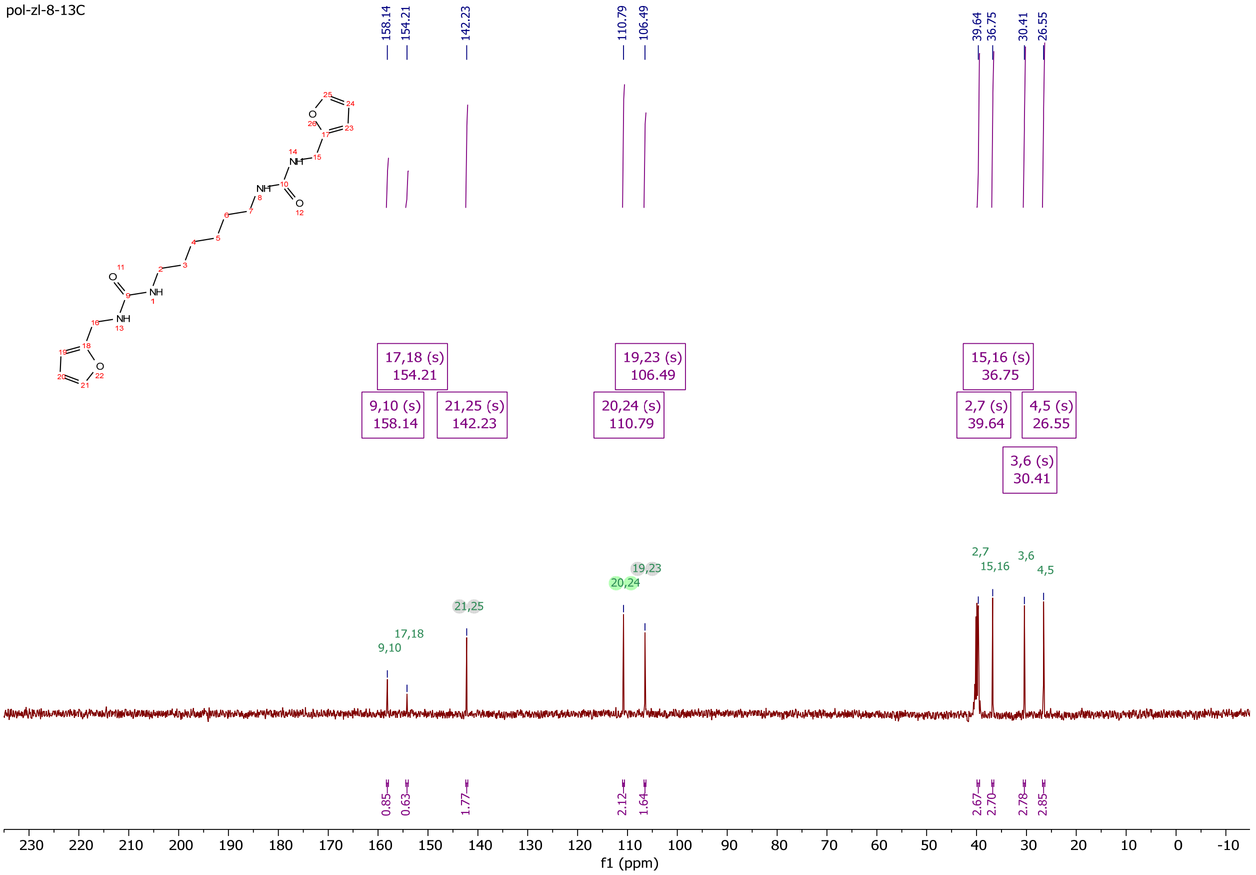

Figure S16 ^{13}C NMR spectrum of FA-H in DMSO-d$_{6}$.

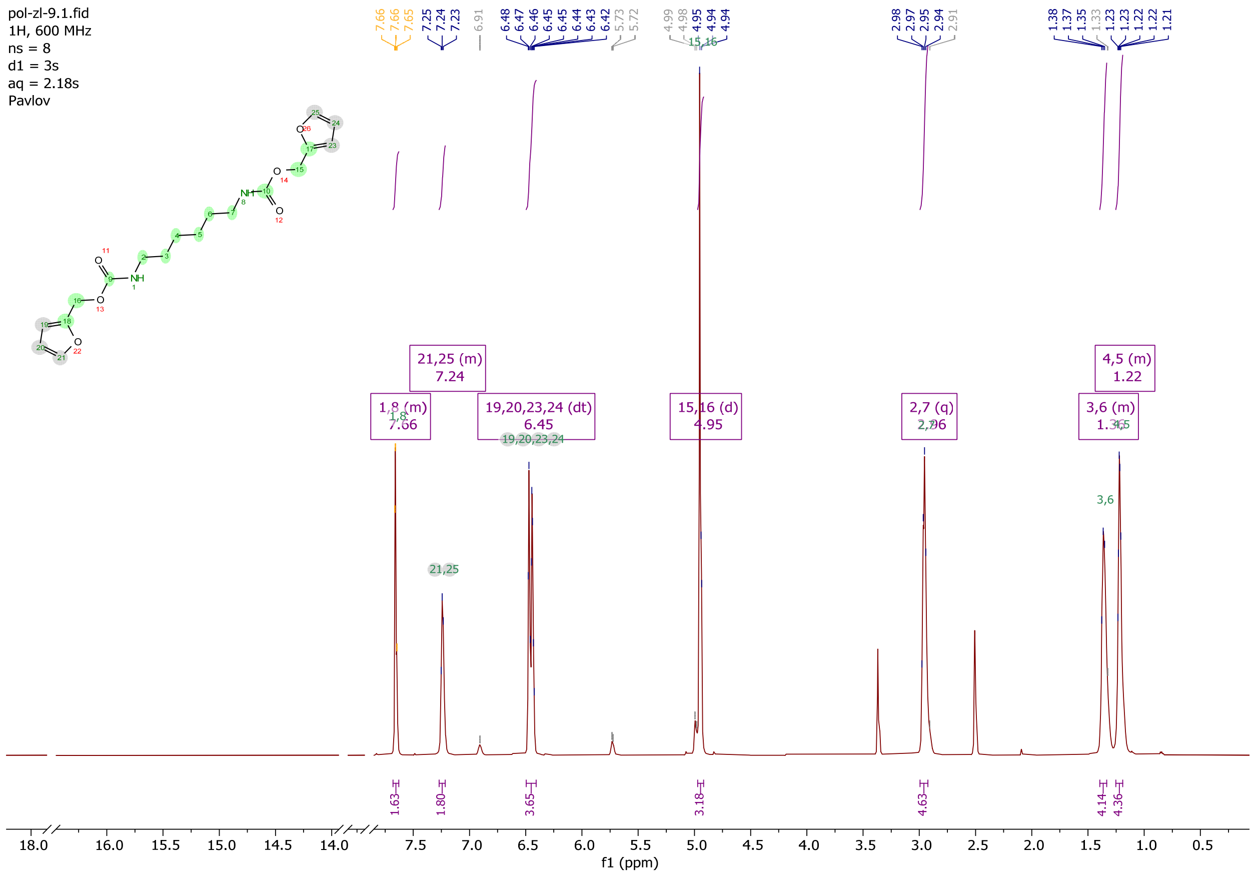

Figure S17 ^{1}H NMR spectrum of FOH-H in DMSO-d^{6}.

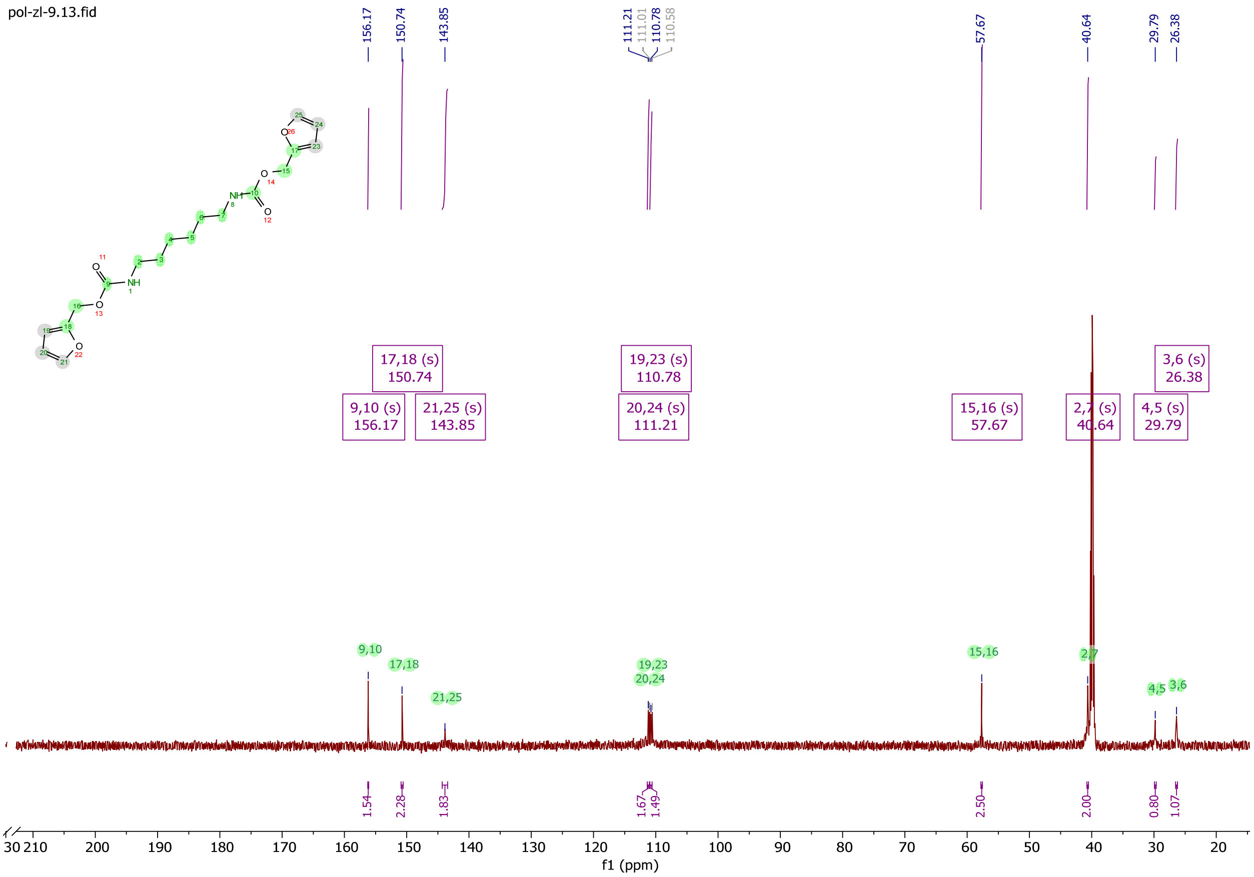

Figure S18 ^{13}C NMR spectrum of FOH-H in DMSO-d$_{6}$.

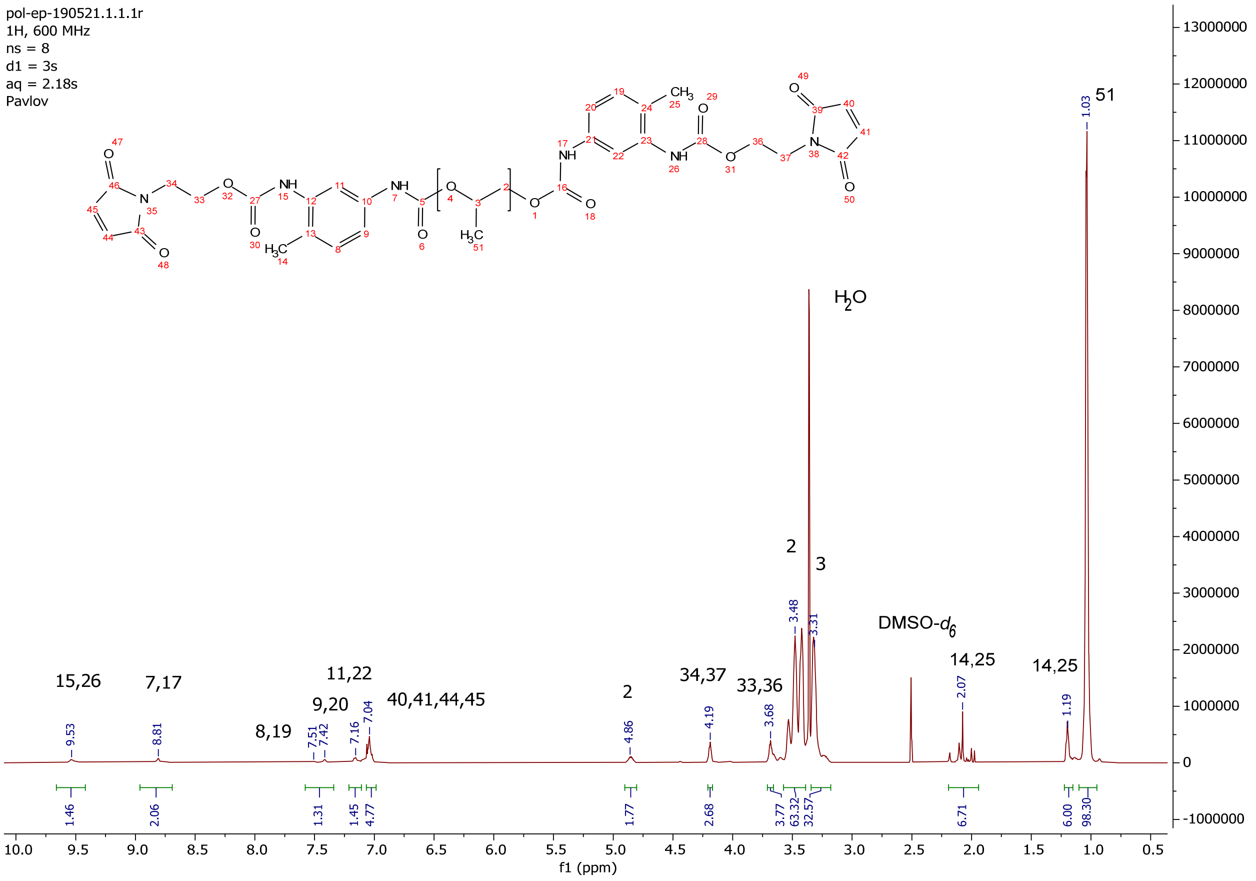

Figure S19 ^{1}H NMR spectrum of prepolymer in DMSO-d^{6}.

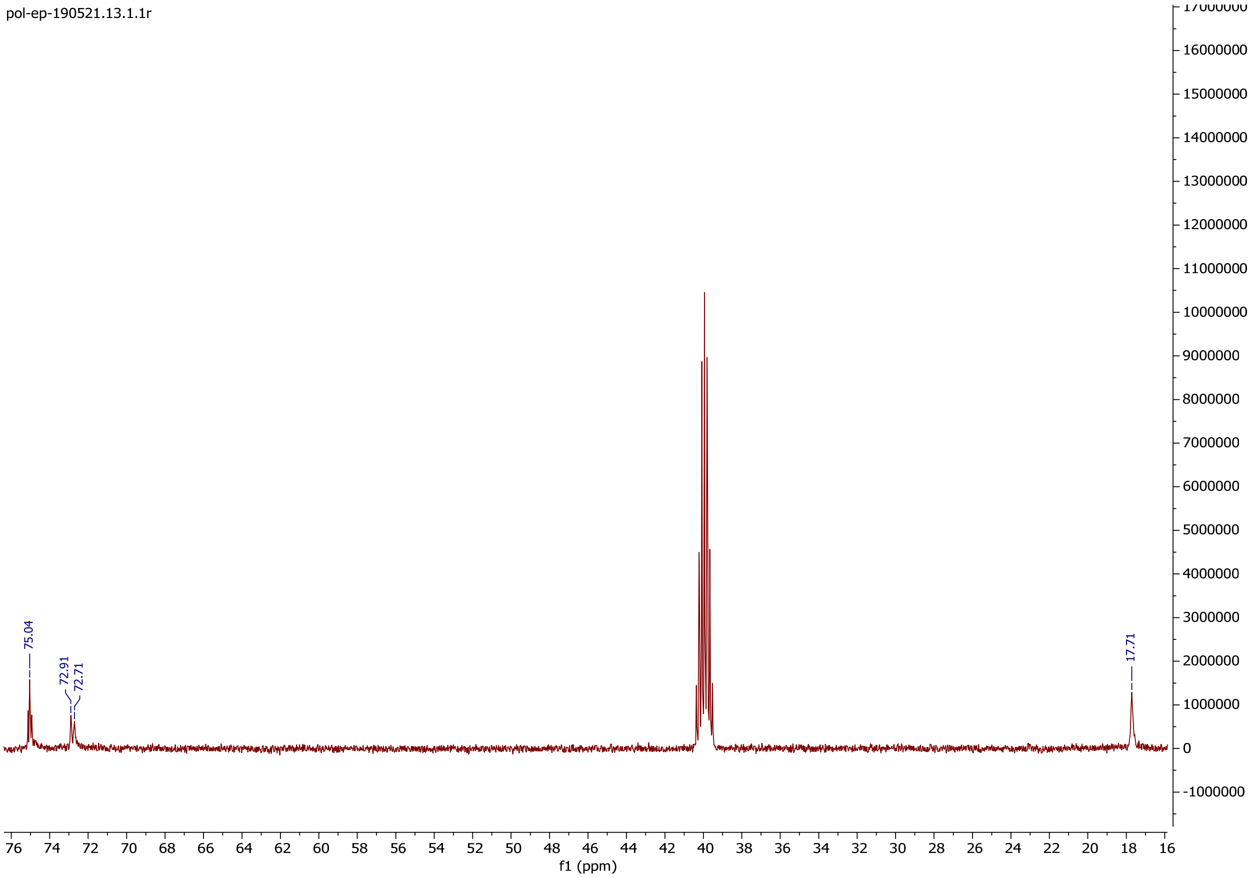

Figure S20 ^{13}C NMR spectrum of prepolymer in DMSO-d$_{6}$.

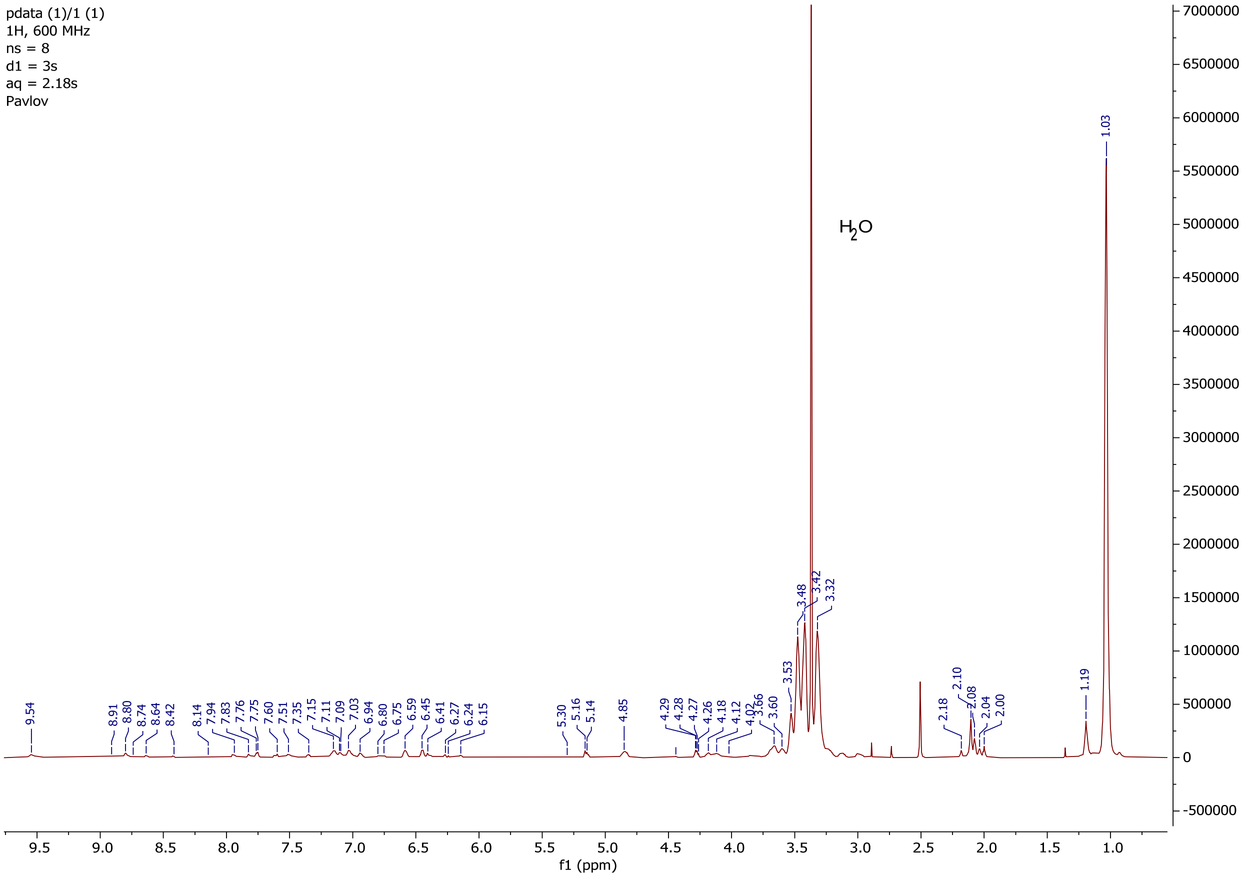

Figure S21 ^{1}H NMR spectrum of PU-DA in DMSO-d^{6}.

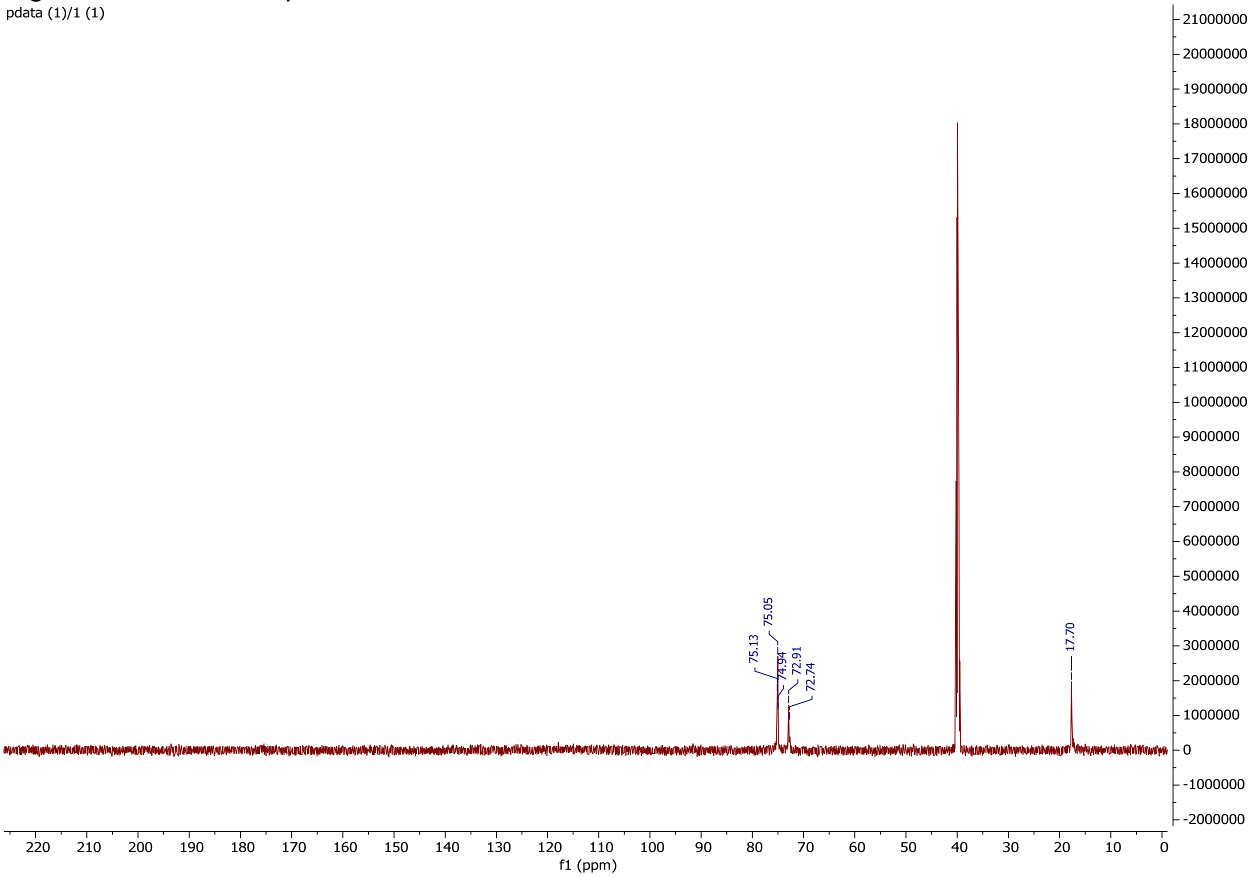

Figure S22 ^{13}C NMR spectrum of PU-DA in DMSO-d^{6}.

3.2 Attenuated Total Reflection-Fourier Transform Infrared Spectroscopy (ATR-FTIR)

ATI-FTIR was performed on Nicolet iS10 spectrometer in the range of 4000 to 650 cm^{-1} on germanium crystal.

3.3 Differential Scanning Calorimetry (DSC)

The thermal behavior was examined by DSC, with a NETZSCH DSC 204 F1 Phoenix within a temperature range of -80 to 180 °C at heating/cooling rates of 10 K min^{-1} in argon atmosphere. A sample weight of about 18 mg was used for measurement.

Table S1. Thermal properties of furan-containing ureas

Sample	Mp		$T_{5\%}$, °C	T_{10}%, °C	T_{max}, °C	Residue, %
	In vacuum*	By DSC				
DFA-T	128	91	201	214	250	8.4
FA-T	285**	231	223	229	286	14.2
FOH-T	104	108	211	218	227	26.2
DFA-M	120	124	201	221	258	26.9
FA-M	-	231-245**	243	254	272	22.6
FOH-M	119**	141	192	211	225	37.7
DFA-H	89	94	245	260	302	1.4
FA-H	206	212	241	257	289	22.6
FOH-H	98	107	227	232	246	11.5

* Measured in sealed capillary.

** With visible decomposition.

FA-M sample did not show any visible signs of melting upon heating to 300 °C in vacuum in sealed capillary.

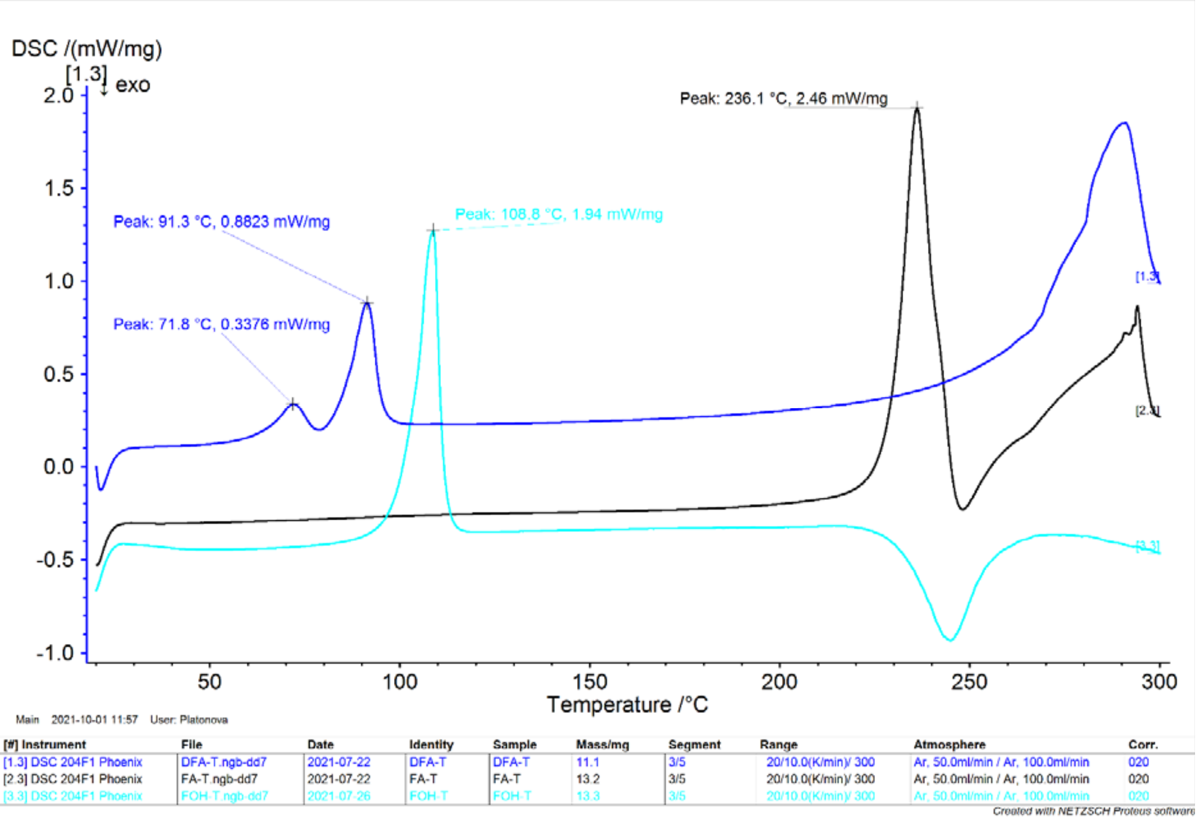

Figure S23 DSC curves of the building blocks of the T-series.

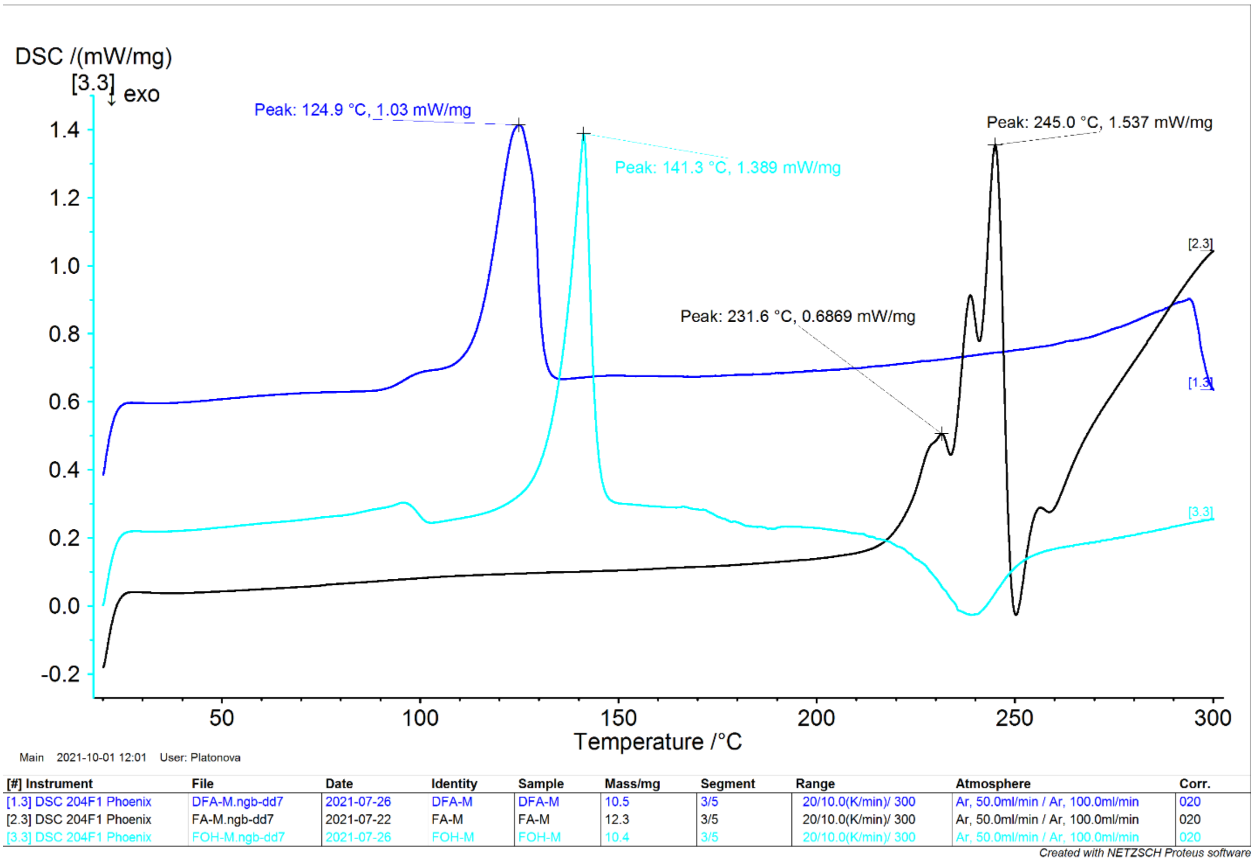

Figure S24 DSC curves of the building blocks of the M-series.

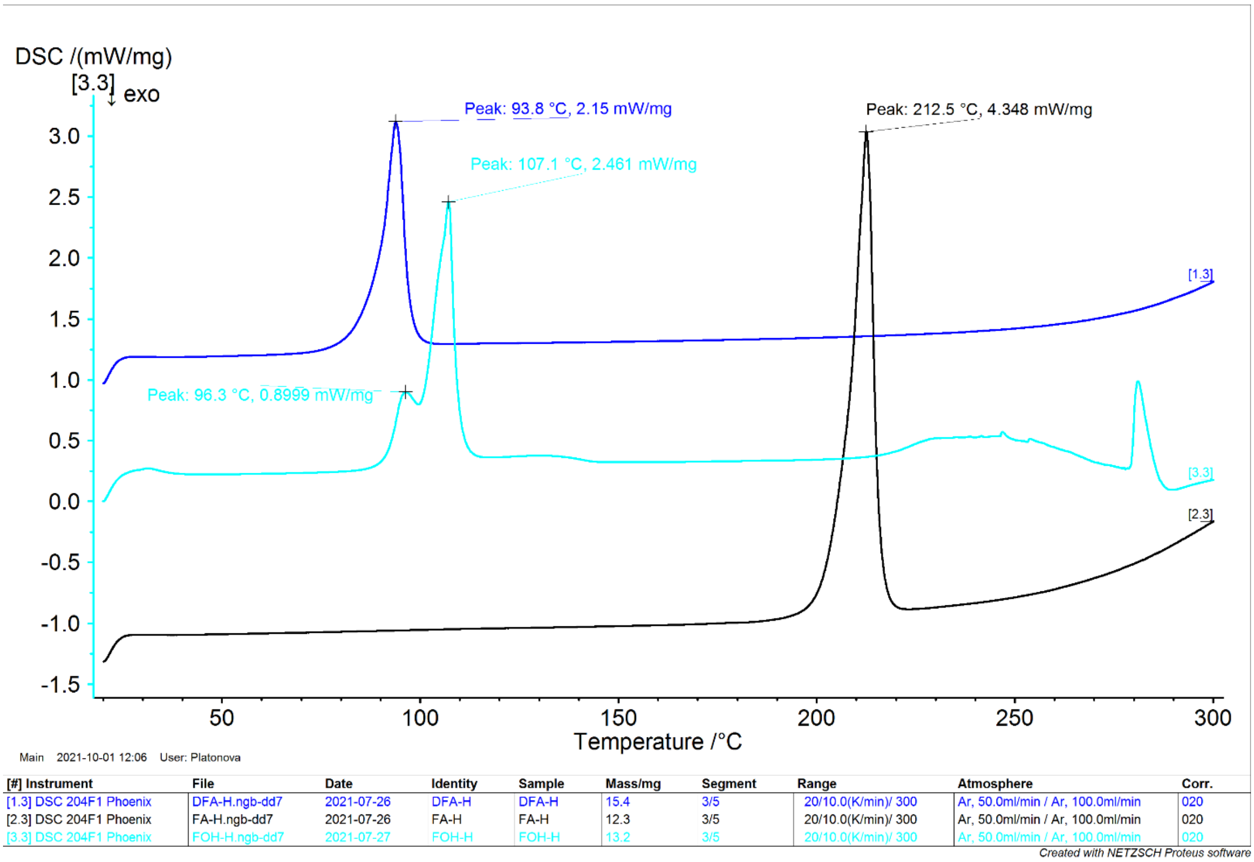

Figure S25 DSC curves of the building blocks of the H-series.

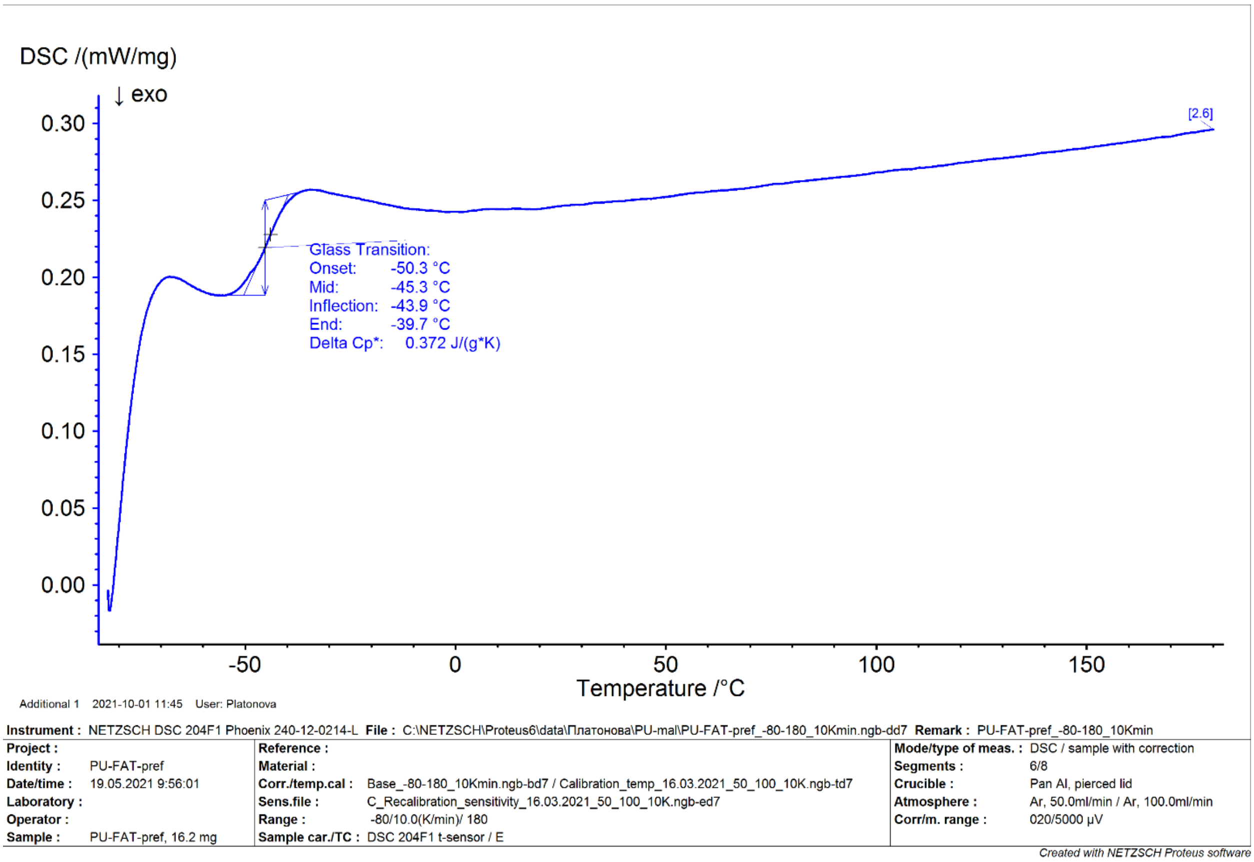

Figure S26 DSC curve of the PU-DA prepolymer.

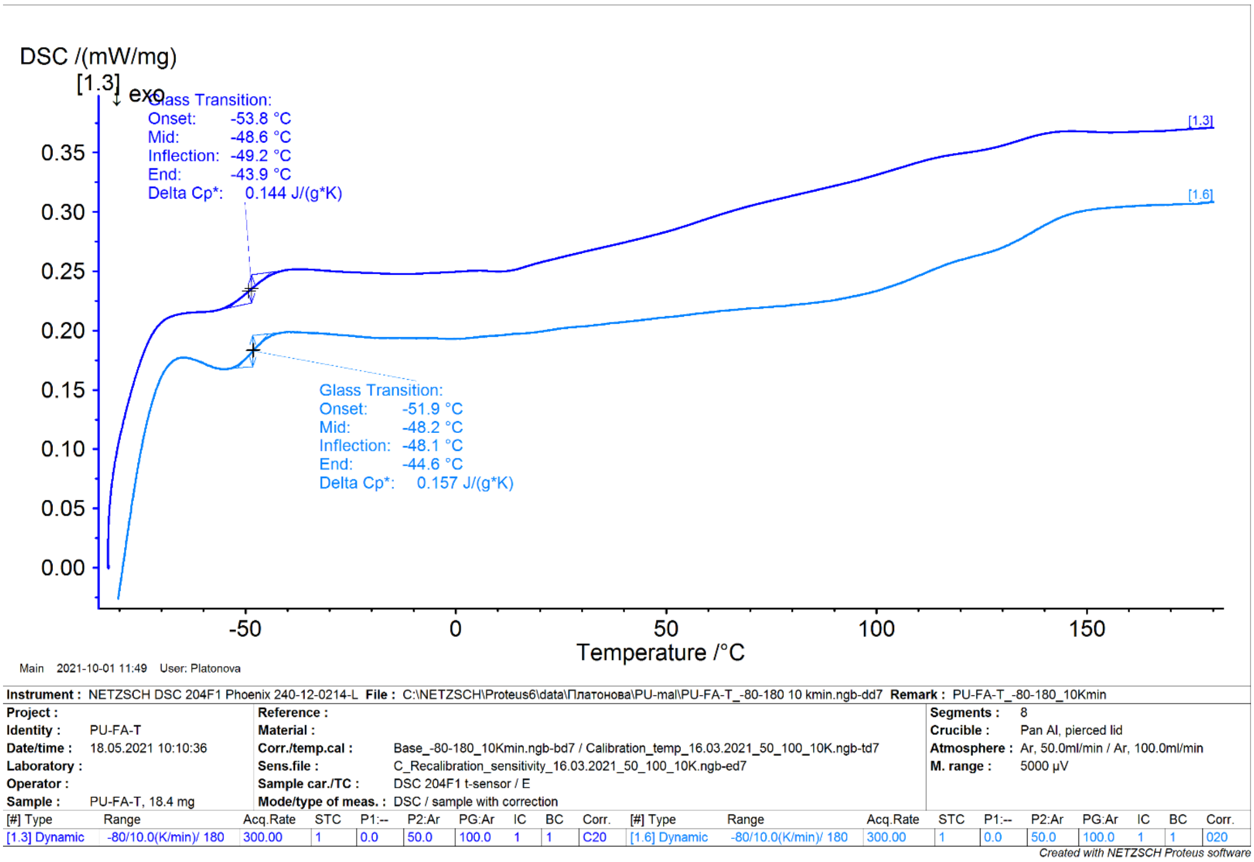

Figure S27 DSC curve of the PU-DA (the first and second heating curve).

3.4 Thermogravimetric Analysis (TGA)

Thermogravimetric analysis (TGA) was performed on NETZSCH TG 209 F1 Libra within a temperature range of 30 to 550 °C at heating/cooling rate of 10 K min^{-1} in an argon atmosphere.

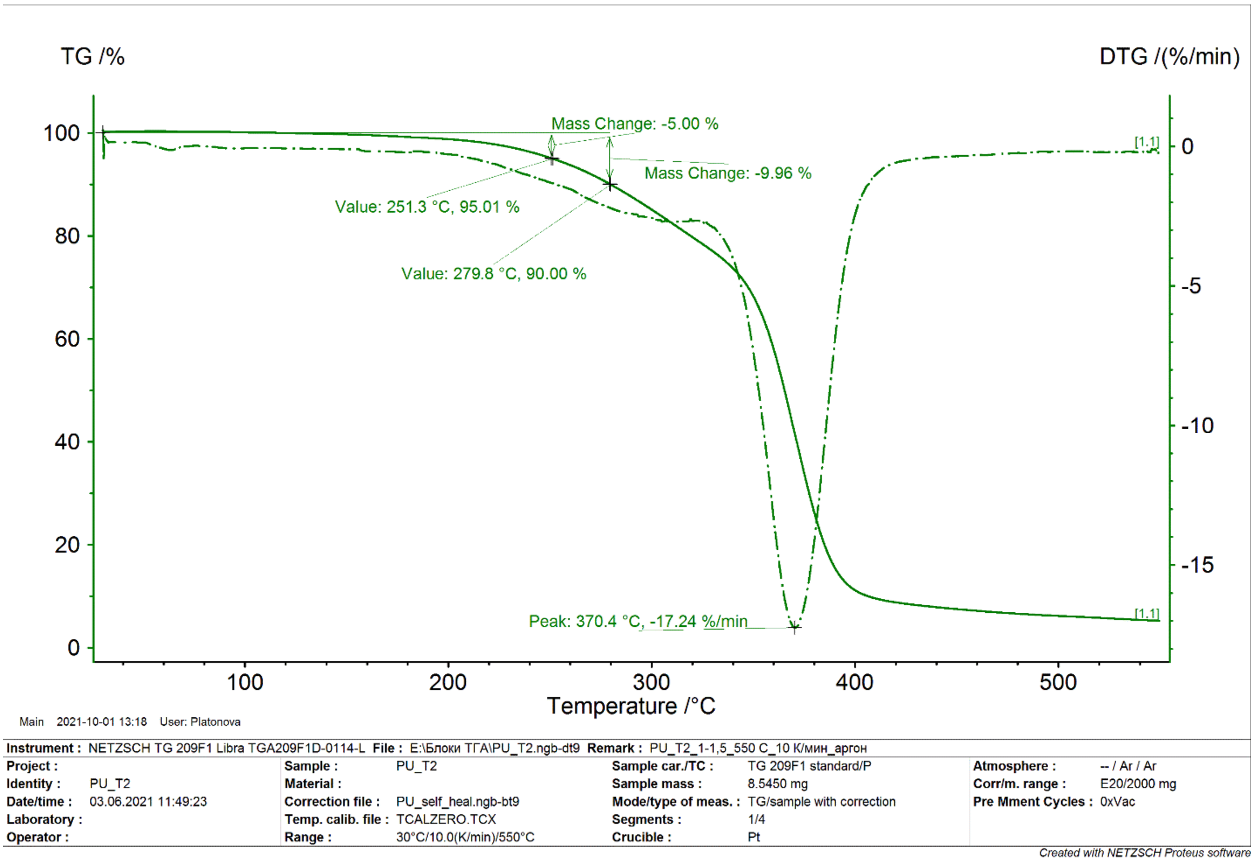

Figure S28 TGA and DTG curves of PU-DA.

3.5 Gel permeation chromatography (GPC)

Gel permeation chromatography (GPC) analyses were performed using a Shimadzu Prominence LC-20 apparatus equipped with a RID 20A refractive index detector and three PSS GRAM analytical columns 100 Å (8.0 × 300 mm^2) linked in series, thermostatted at 40 °C, and using N,N-dimethylformamide (DMF) containing 0.5 g/l LiBr as the mobile phase at a flow rate of 1.0 mL min^{-1}. Molecular weights were estimated against polymethylmethacrylate (PMMA) standards.

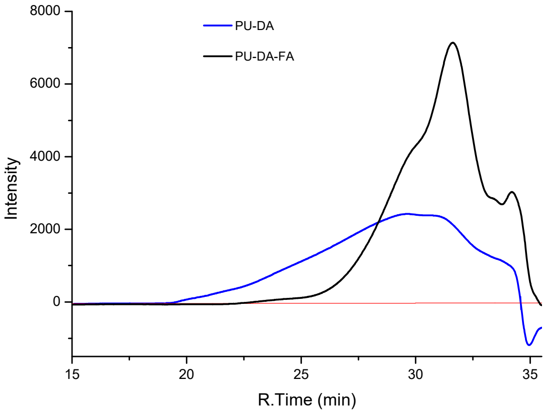

Figure S29 GPC chromatograms of PU-DA and PU-DA-FA.

3.6 Quantification of self-healing process efficiency

Mechanical properties (σ_t, E, ε, σ_t (ε) were determined from the uniaxial tensile strength test. Dogbone-shaped samples were cut from film using a special blade (length 15 mm, width 2 mm, see Figure S29 and Table S2 in the Supplementary Materials) and hand press. Film thickness was from 1.5 to 2.0 mm. The samples were stretched on a Zwick Roell Z2.5 universal testing machine at a 150 mm/min loading speed. An applied load P and a strain ε were recorded as loading diagrams. Further, the stress at break σ_t, the elongation ε, and the elastic modulus E were calculated from the diagrams.

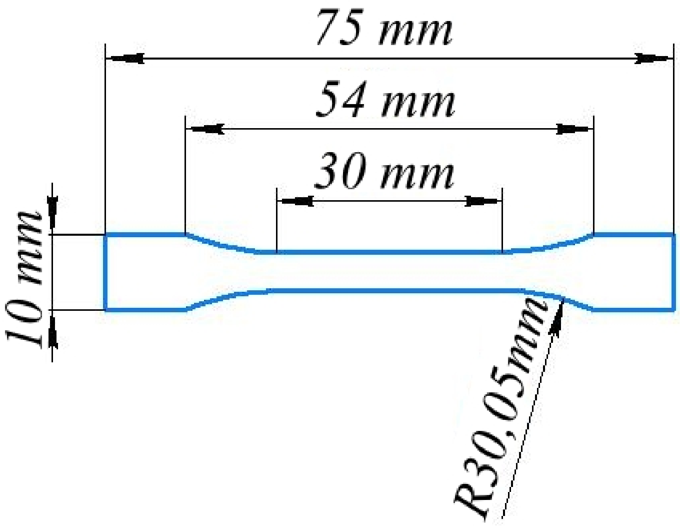

Figure S30 Shape of tensile strength samples of PU-DA.

Table S2. Tensile test parameters.

Preload, N	1.2
Preload speed, mm/min	50
Test load speed, mm/min	150/800

Value of preload was chosen in accordance with accuracy of the force sensor (± 1 N) for getting rid of compressive stresses in material, which occur after installation of test samples in the clamps of tensile-machine).

For quantification of self-healing process efficiency, the measurement of elastic modulus and tensile strength was performed. As a control measurement, five samples from the first group were tested for tension. The samples from the second group were damaged with a blade in the middle. Damaged samples were subjected to thermal treatment: 2 h at 120 C and 4 days at 60 °C. After completion of the healing process, the samples from the second group were tested for tension in the same way as the first (control) group. For quantification of elastic modulus in each group of samples, mean load curve was used (average curve for all samples of the group).

On that basis, we could assume that in this strain range, the material had only elastic deformations and Hooke's law can be applied. For each group of samples, the elastic modulus was quantified using the least squares method. The fit function is linear:

$$\sigma(\varepsilon) = E\varepsilon$$

As a result, for each group of samples, the elastic modulus was calculated. We propose to use the modulus recovery factor [η_E, eq. (1)] and the strength recovery factor [η_σ, eq. (2)][4] for quantification of the efficiency of self-healing process:

$$\eta_E = \left(1 - \frac{E_{origin} - E_{healing}}{E_{origin}}\right) \cdot 100\% \quad (1)$$

$$\eta_\sigma = \left(1 - \frac{\sigma_{origin} - \sigma_{healing}}{\sigma_{origin}}\right) \cdot 100\% \quad (2)$$

where E_{origin} is the elastic modulus of original samples (MPa), $E_{healing}$ is the modulus of healed samples (MPa), σ_{origin} and $\sigma_{healing}$ (MPa) are the strength values of original and healed samples, respectively.

References

[1] Y.L. Gol'dfarb, é.G. Ostapenko, V.G. Vinogradova, A.N. Zverev, A.V. Polyakov, A.I. Yanovskii, D.S. Yufit, Y.T. Struchkov, Synthesis, structures, and redox properties of some new dithiocarbamates that include a heteroaromatic ring, Chem. Heterocycl. Compd. 23(7) (1987) 740-747.

[2] N. Salewska, M.J. Milewska, Efficient Method for the Synthesis of Functionalized Basic Maleimides, J. Heterocycl. Chem. 51(4) (2014) 999-1003.

[3] S. Yu, R. Zhang, Q. Wu, T. Chen, P. Sun, Bio-Inspired High-Performance and Recyclable Cross-Linked Polymers, Adv. Mater. 25(35) (2013) 4912-4917.

[4] L. Feng, Z. Yu, Y. Bian, J. Lu, X. Shi, C. Chai, Self-healing behavior of polyurethanes based on dual actions of thermo-reversible Diels-Alder reaction and thermal movement of molecular chains, Polymer 124 (2017) 48-59.
